# The phenyl vinyl ether–methanol complex: a model system for quantum chemistry benchmarking

**DOI:** 10.3762/bjoc.14.140

**Published:** 2018-07-02

**Authors:** Dominic Bernhard, Fabian Dietrich, Mariyam Fatima, Cristóbal Pérez, Hannes C Gottschalk, Axel Wuttke, Ricardo A Mata, Martin A Suhm, Melanie Schnell, Markus Gerhards

**Affiliations:** 1 Fachbereich Chemie & Research Center Optimas, Technische Universität Kaiserslautern, Erwin-Schrödinger-Str. 52, D-67663 Kaiserslautern, Germany; 2 Max Planck Institute for the Structure and Dynamics of Matter, Luruper Chaussee 149, D-22761 Hamburg, Germany; 3 Deutsches Elektronen Synchrotron (DESY), Notkestrasse 85, D-22607 Hamburg, Germany; 4 Institut für Physikalische Chemie, Georg-August-Universität Göttingen, Tammannstrasse 6, D-37077 Göttingen, Germany; 5 Institute of Physical Chemistry, Christian-Albrechts-Universität zu Kiel, Max-Eyth-Strasse 1, D-24118 Kiel, Germany

**Keywords:** dispersion interactions, IR spectroscopy, quantum-chemical calculations, rotational spectroscopy, structure determination, weak hydrogen bonds

## Abstract

The structure of the isolated aggregate of phenyl vinyl ether and methanol is studied by combining a multi-spectroscopic approach and quantum-chemical calculations in order to investigate the delicate interplay of noncovalent interactions. The complementary results of vibrational and rotational spectroscopy applied in molecular beam experiments reveal the preference of a hydrogen bond of the methanol towards the ether oxygen (OH∙∙∙O) over the π-docking motifs via the phenyl and vinyl moieties, with an additional less populated OH∙∙∙P(phenyl)-bound isomer detected only by microwave spectroscopy. The correct prediction of the energetic order of the isomers using quantum-chemical calculations turns out to be challenging and succeeds with a sophisticated local coupled cluster method. The latter also yields a quantification as well as a visualization of London dispersion, which prove to be valuable tools for understanding the role of dispersion on the docking preferences. Beyond the structural analysis of the electronic ground state (S_0_), the electronically excited (S_1_) state is analyzed, in which a destabilization of the OH∙∙∙O structure compared to the S_0_ state is observed experimentally and theoretically.

## Introduction

The balance of different noncovalent interactions is crucial for chemical and biochemical processes as it controls molecular recognition and aggregation [1–6]. In order to gain a deeper understanding of these processes, knowledge on exact structural arrangements and the respective role of different intermolecular forces such as electrostatic, dispersion and induction forces is needed. Thus, experimental examination as well as the precise prediction of a preferred molecular docking site for different molecules is of crucial importance. Despite the remarkable progress made in experiments and theory/computational chemistry, there is still a need for improvement and benchmarking [7].

Many aromatic solute–solvent complexes have been studied in the gas phase (cf. [8–10] and references therein). Studied systems involving methanol as attached solvent molecule include the works on benzene–methanol clusters by the Zwier group [11] and on fluorobenzene–methanol clusters by the Brutschy group [12], to mention only two examples. Complexes of aromatic ethers with polar solvent molecules are of special interest due to the presence of different competing hydrogen bond acceptor sites. An extensive study on diphenoxyethane–water clusters was performed by the Zwier group [13–15] including studies in the excited S_1_ and S_2_ states. Concerning aggregates of aromatic ethers with alcohols, there is a work of Pietraperzia et al. [16] on the anisole–phenol complex in which an OH∙∙∙O structure was identified. In a systematic study by the Suhm group on complexes of anisole derivatives with methanol, a balance between OH∙∙∙O and OH∙∙∙π structures being very sensitive to the substitution pattern at the anisole moiety was identified [17–18]. In previous multi-spectroscopic studies by the Schnell, Suhm and Gerhards groups on diphenyl ether (DPE)–solvent complexes [19–22], the influence of different attached solvent molecules on the structural preference was compared. It could be shown that the balance between OH∙∙∙π- and OH∙∙∙O-bound structures is very sensitive to the size of the attached alcohol. Torsional balances in solution have been used to probe aromatic OH∙∙∙π interactions and to show that these interactions remain important at room temperature [23].

In such aromatic solute–solvent systems, one frequently encounters hydrogen bonds formed towards oxygen or nitrogen lone pairs, or R–H∙∙∙π binding motifs (R = O, N, C, S,…) involving aromatic π systems. Less often, R–H∙∙∙π bound complexes are found involving nonconjugated, localized C=C double bonds. Exceptions include the ethene–methanol complex [24] as well as bulky olefin–*tert*-butyl alcohol complexes [25] investigated by jet FTIR spectroscopy. The observed OH stretching red-shifts compared to the free alcohols are small, indicating a comparatively weak hydrogen bond, which is also reflected in calculated binding energies [24–25].

With the herein presented work, we now extend our overall multi-spectroscopic study to mixed aromatic olefinic ethers: in the case of phenyl vinyl ether (PVE), there is an ethenyl moiety replacing one of the phenyl rings compared to DPE. This introduces a localized π system along with the delocalized phenyl π system as hydrogen bond acceptor sites. Thereby, the complexity of the system is increased, as now three qualitatively different basic binding motifs have to be regarded instead of only two for DPE. This also provides an enhanced challenge for theory, with no clear preference for one of the motifs to be expected. As shown, e.g., in the case of DPE–*t*-BuOH [20], there is a need for benchmarking systems in order to improve and develop better theoretical approaches especially for non-covalently bound complexes. The study on PVE–MeOH is meant to present a further benchmark system, probably even more challenging than DPE–*t*-BuOH.

For an experimental elucidation of structural arrangements and energetic preferences, investigations on a molecular level are required on isolated molecular aggregates, allowing for an ideal comparison with gas phase calculations. This can be achieved by molecular beam experiments, which can be combined with a variety of spectroscopic techniques. For our multi-spectroscopic studies, we utilize FTIR spectroscopy, mass- and isomer-selective IR/UV techniques (IR/R2PI, for methodical developments, cf., e.g., [8,26–29] and UV/IR/UV spectroscopy, cf., e.g., [30–43]) and chirped-pulse Fourier transform microwave (CP-FTMW) spectroscopy. Comparing spectroscopic results with quantum-chemical calculations is often mandatory for the interpretation of experiments. Furthermore, such comparison enables a critical evaluation of the approximations used, comparing the relative stability of different binding motifs.

In this paper, the first structural investigation on the complex of phenyl vinyl ether with methanol is presented. An established multi-spectroscopic approach [19–20] is used, coupling FTIR, IR/UV and microwave spectroscopy with theoretical treatments including dispersion-corrected density functional theory (DFT-D3) [44–45], spin-component-scaled approximated coupled cluster-singles-doubles (SCS-CC2) [46] as well as explicitly correlated local coupled cluster theory (LCCSD(T0)-F12) [47] calculations, the latter allowing for a quantification and visualization of London dispersion interactions [48]. The aim of the presented study is the unambiguous experimental identification of the preferred binding site of a first methanol solvent molecule to the multivalent hydrogen bond scaffold of phenyl vinyl ether, followed by a classification of theoretical methods in terms of success or failure to predict this preference. Visualization of possible reasons for the subtle preference is a valuable additional asset.

## Experimental Setup

### FTIR setup

For the FTIR experiments, the so-called filet-jet setup, as described in detail in [49], was used. In this setup, the scans of a Bruker IFS 66 v/s spectrometer (80 kHz, resolution 2 cm^−1^) are synchronized to a pulsed supersonic expansion through a 600 × 0.2 mm^2^ slit nozzle. Using two separate cooled saturators, low concentrations (<0.1%) of PVE (Sigma-Aldrich, 97%, used as purchased) and methanol (Sigma-Aldrich, ≥99.8%, used as purchased) were added to the carrier gas helium (Linde, 99.996%) and premixed at a pressure of 0.75 bar in a 67 L reservoir before being expanded through the slit nozzle. The pulsed operation with waiting times of 30–90 s between 150 ms long pulses combined with a buffer volume of 12–23 m^3^ and a pumping capacity of 500–2500 m^3^/h resulted in background pressures of less than 0.1 mbar before expansions. This facilitated measurements of clusters of methanol and PVE in the zone of silence of the expansion at an average distance of 10 mm to the nozzle. A calcium fluoride beam splitter, lenses and windows were used in combination with a 150 W tungsten filament and an optical filter (4200–2450 cm^−1^) to maximize the signal-to-noise ratio in the OH stretching range of the vibrational spectra. For the final spectra, 150 to 775 pulses were co-added to further improve signal-to-noise.

### IR/UV setup

The experimental setup for the IR/UV experiments is described in detail elsewhere [29,50], thus only a brief description is given here. All experiments were carried out in a molecular beam apparatus consisting of a differentially pumped linear time-of-flight (TOF) mass spectrometer with a pulsed valve (Series 9 and pulse driver Iota One, General Valve, 500 µm orifice) for skimmed jet expansion. PVE was synthesized according to the procedure reported in [51] (cf. Supporting Information File 1 for details). MeOH (Sigma-Aldrich, ≥99.7%) and PVE were both supplied via separate cooled reservoirs (approx. −8 °C and −13 °C, respectively) and co-expanded with the carrier gas neon (2.5–3.0 bar).

For the one- and two-color R2PI, the IR/R2PI and the UV/IR/UV experiments up to three tunable nanosecond laser systems were necessary, including two independent UV laser systems and one IR laser system. The UV laser radiation is obtained via second harmonic generation in a BBO crystal using the output of a dye laser (Cobra-Stretch and PrecisionScan, Sirah). They are pumped by the second harmonic (532 nm) of a Nd:YAG laser (SpitLight 600 and SpitLight 1000, Innolas). The IR laser radiation in the range of 3520–3750 cm^−1^ is generated by difference frequency mixing (DFM) in a LiNbO_3_ crystal using the fundamental (1064 nm) of a seeded Nd:YAG laser (Quanta-Ray Pro-230, Spectra-Physics) and the output of a further dye laser (PrecisionScan, Sirah), which is pumped by the second harmonic (532 nm) of the same Nd:YAG laser. Amplification of the resulting IR radiation is obtained by an optical parametric amplification (OPA) process in a further LiNbO_3_ crystal using the output of the DFM process and the fundamental (1064 nm) of the Nd:YAG laser.

For the IR/R2PI spectra, the IR laser was fired 50 ns prior to the UV excitation laser, whereas for the UV/IR/UV spectra the IR laser was fired 2.0–3.0 ns after the UV excitation laser. The time delay between UV excitation and ionizing laser was 4.0–4.5 ns.

### CP-FTMW setup

The rotational spectroscopy measurements were performed with the Hamburg chirped-pulse Fourier transform microwave (CP-FTMW) spectrometer COMPACT covering the 2–8 GHz frequency range, which has been described in detail in [52]. The molecules were seeded into a supersonic expansion with neon as the carrier gas by using a pulse nozzle (Parker General Valve, Series 9, 0.9 mm diameter orifice) equipped with a heatable reservoir close to the valve orifice, operating at 8 Hz. PVE was synthesized as described above and used without further purification.

The liquid sample was held in the reservoir at room temperature, which resulted in sufficient vapor pressure (standard boiling point of about 155 °C) for recording the rotational spectrum. MeOH was kept in a separate reservoir. PVE–MeOH clusters were generated by first flowing the carrier gas (neon) through the reservoir containing methanol that was external to the chamber, followed by picking up PVE vapor. After supersonic expansion into vacuum using neon at 3 bar, the molecular jet was polarized with a 4 µs chirp spanning 2–8 GHz. The chirp was generated with an arbitrary waveform generator, amplified to 300 W with a traveling wave tube amplifier, and transmitted into the vacuum chamber via a horn antenna. Following excitation, 40 µs of the free induction decay (FID) of the macroscopic ensemble of polarized molecules was recorded. The fast frame capability [53] of the Tektronix DPO 71254C was used in which eight consecutive excitation chirps, each followed by 40 µs during which the FID could be collected, were recorded and averaged. This resulted in an effective repetition rate of 64 Hz.

For the spectrum of the PVE–MeOH dimer, 3 million FIDs were co-added. A resulting signal-to-noise ratio of about 500:1 to 600:1 for the stronger transitions of the dominant complex allowed us to determine the positions of the carbon atoms with respect to the center of mass of the overall complex (see below) exploiting the presence of ^13^C isotopologues in natural abundance and using the Kraitchman approach [54]. Fourier transformation of the averaged time domain FID, recorded at point spacings of 10 ps, resulted in a frequency domain rotational spectrum with frequency resolution of 25 kHz.

The assignment was performed with the program JB95 [55], then the fits to an asymmetric-rotor Hamiltonian were performed using SPFIT/SPCAT. The experimental results were complemented by and compared with the results of electronic structure calculations. B3LYP-D3/aug-cc-pVTZ calculations were performed using the Gaussian 09, rev. D.01 program suite [56] to guide the assignment.

### Computational Methods

Various input structures for the PVE–MeOH complex were generated by using the MMFF94s force field [57] as implemented in Avogadro [58]. Afterwards, geometry optimizations were performed by applying the Berny optimization algorithm of Gaussian 09 [56] with energies and gradients obtained from Turbomole 7.0 [59]. The DFT functional B3LYP with Grimme's two-body D3 corrections and Becke–Johnson damping [45] was used in combination with the def2-TZVP basis set based on the documented performance of this level of theory for the similar diphenyl ether–methanol system [19]. Furthermore, the obtained structures were re-optimized with the SCS-CC2 method using the def2-TZVP basis set, both in the electronic ground (S_0_) and first excited state (S_1_). The ricc2 module in Turbomole 7.0 requires an auxiliary Coulomb fitting basis set (cbas) for the resolution-of-identity approximation (RI) for which def2-TZVP-cbas was chosen [60]. All obtained geometries were confirmed as minima by harmonic frequency calculations.

In order to evaluate the relative stability of the different conformers found on the potential hypersurface, density fitted explicitly correlated local coupled cluster with singles and doubles excitations and perturbative triples (DF-LCCSD(T0)-F12) calculations were carried out [47]. In order to converge the energies relative to the one particle basis, the VTZ-F12 and VQZ-F12 basis sets [61–62] were used together with a Schwenke style basis set extrapolation, as proposed in [63]. The orbitals were Pipek–Mezey [64] localized and orbital domains determined by natural population analysis with a threshold of TNPA = 0.03 [65]. Defaults were used for the pair classification, with all pairs included in the F12 treatment. Furthermore, the intermolecular pairs were classified as strong (meaning that they were treated at the highest level of theory). The latter method will be denoted as LCCSD(T0)-F12/CBS[T:Q]. In all correlated calculations the 1s electrons were removed from the treatment (frozen-core approximation). Furthermore, we analyzed the relative impact of dispersion interactions in the different complexes through a local orbital analysis of the CCSD (connected) doubles energy terms. The latter discussion is complemented with dispersion interaction density (DID) plots [48]. The coupled cluster calculations were carried out with Molpro 2015.1 [66].

## Results and Discussion

### Theoretical results

**Figure 1:**
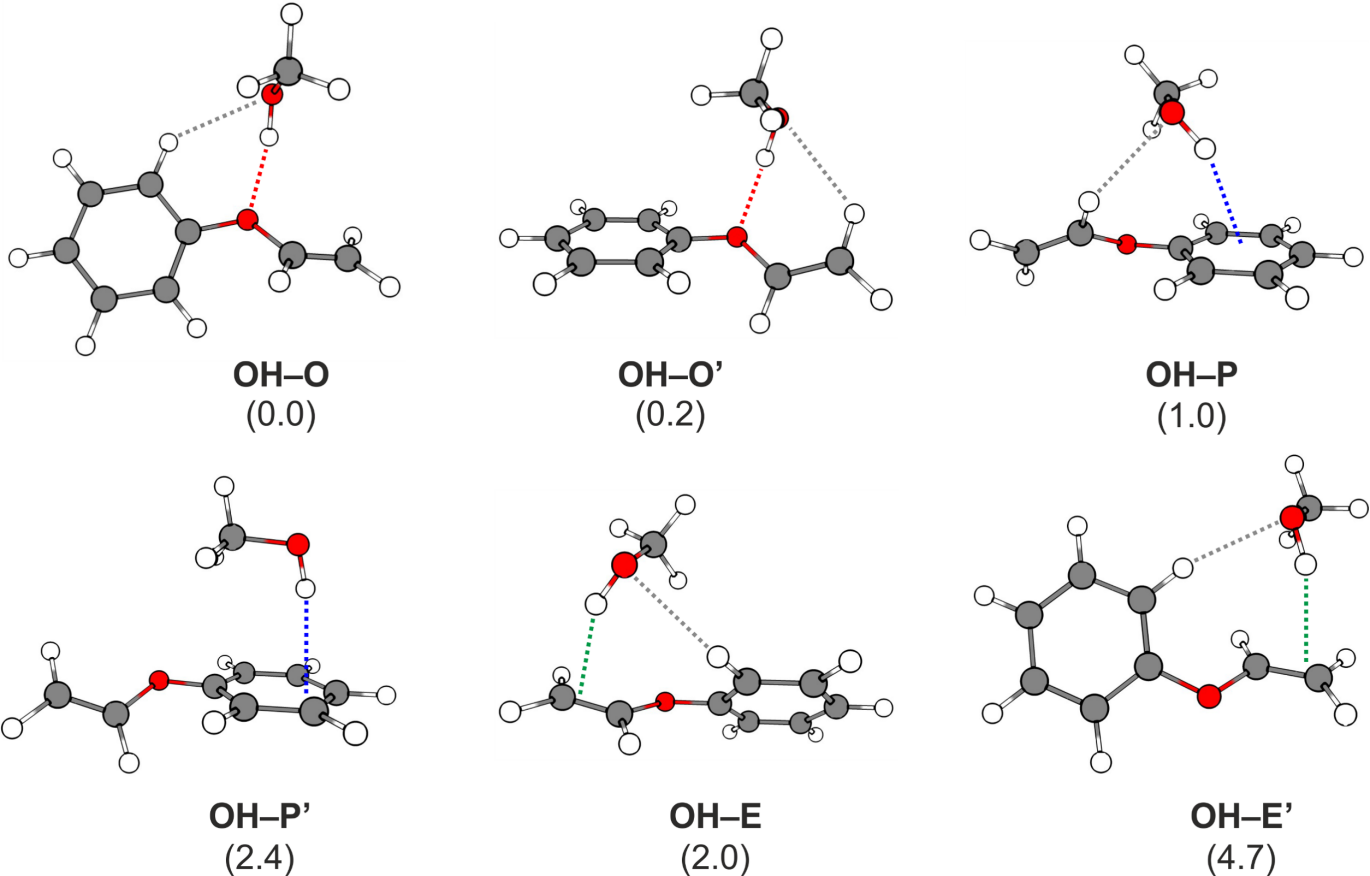
Minimum structures of the most stable PVE–MeOH dimers obtained at the B3LYP-D3(BJ)/def2-TZVP level; dashed colored lines indicate the different primary docking motifs, dashed gray lines illustrate secondary CH–O contacts; values in parentheses correspond to the relative, zero-point-corrected energies *E*_0,rel_ with respect to the OH–O isomer, calculated at the LCCSD(T0)-F12/CBS[T:Q]//B3LYP-D3/def2-TZVP level of theory (cf*.* Table 1).

In contrast to the already studied diphenyl ether–alcohol clusters [19–20,22], phenyl vinyl ether offers three different binding sites for possible interactions with small solvent molecules: the ether oxygen, the phenyl ring and the vinyl moiety. Since both the phenyl ring and the vinyl moiety interact with the solvent via a π cloud, preferred binding sites are indicated using the following nomenclature: P (phenyl) and E (ethenyl), respectively. The optimizations using B3LYP-D3(BJ)/def2-TZVP yield six different structures, representing each binding motif with two isomers (cf. Figure 1).

In order to verify the structures of the complexes, a second level of theory was applied, namely SCS-CC2/def2-TZVP. Similar minima were found in the latter calculations, confirming the rich variety of binding motifs. However, distinct differences were found between the two methods: While B3LYP-D3 predicted the OH–E conformer as the most stable complex SCS-CC2 gave OH–P as the lowest minimum (cf. Table S1 in Supporting Information File 1). This is in contrast to our results of diphenyl ether–alcohol clusters [19–20,22], where both computational levels predicted the same energetic order of the isomers. The two structures correspond to quite different docking positions, reflecting well the demanding test this system imposes on quantum chemical methods. Several minima are separated by energy differences of 1 kJ/mol or less. The complete energetic analysis at both levels of theory is presented in Supporting Information File 1 (cf. Tables S1 and S2). The reasons behind the discrepancies are manifold, ranging from the method to the small basis set used. In order to obtain a more reliable theoretical prediction, LCCSD(T0)-F12/CBS[T:Q] calculations were carried out on top of the DFT-optimized geometries. The results are presented in Table 1, with and without zero-point vibrational energy (ZPVE) corrections.

**Table 1:** Comparison of different structures for PVE–MeOH dimers in the S_0_ state with LCCSD(T0)-F12/CBS[T:Q]//B3LYP-D3/def2-TZVP electronic energies *E*_rel_ and B3LYP zero-point corrected energies *E*_0,rel_ relative to the minimum OH–O structure. The scaled wavenumbers 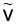 of the OH-stretching vibration together with the respective IR intensity *I* are presented for two levels of theory: B3LYP-D3 (scaling factor: 0.9600) and SCS-CC2/def2-TZVP (scaling factor: 0.9635).

	*E*_rel_ [kJ/mol]	*E*_0,rel_ [kJ/mol]	B3LYP-D3		SCS-CC2	

			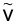 [cm^−1^]	*I* [km/mol]	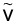 [cm^−1^]	*I* [km/mol]

OH–O	0.0	0.0	3597	219	3619	160
OH–O’	−0.3	0.2	3600	193	3621	144
OH–P	1.4	1.0	3619	112	3631	67
OH–P’	3.9	2.4	3631	127	3636	110
OH–E	1.5	2.0	3567	187	3607	121
OH–E’	4.8	4.7	3567	197	3606	128

The coupled cluster results show a clear energetic preference for the OH–O and OH–O’ isomers. Observing the intermolecular contacts, which may or may not be designated as weak hydrogen bonds but are expected to stabilize the complexes, the main difference between the two structures is a phenyl vs ethenyl CH to methanol O contact (cf. dashed gray lines in Figure 1). Both are separated by only a few tenths of a kJ/mol, which is within the error of the method used (considering that the coupled cluster expansion is truncated at triples excitations and the neglect of core-valence correlation effects, which should be the largest sources of error along with the harmonic B3LYP ZPVE error). It also confirmed the subtle difference between the six conformers, with an energy span of approximately 4–5 kJ/mol (≈1 kcal/mol, the commonly accepted definition of chemical accuracy) among all structures.

Also featured in Table 1 are the computed O–H stretch fundamentals together with the IR intensity at the two different levels of theory used in the optimizations. The frequencies were scaled according to the experimental value of the OH–π isomer of DPE–MeOH [19]. Based on the computational results, the vibrational spectral signals of the OH–O and OH–O’ isomers will be extremely hard to distinguish, as they lie less than 3 cm^−1^ apart, with very similar intensities. The same can be asserted for the less stable OH–E and OH–E’ structures. This is not surprising, given the similarities of the OH binding pattern for both sets of structures.

In order to gain further insight into the energetic order of the different isomers, we conducted an analysis of the dispersion interactions present in the system by decomposing the CCSD energy terms obtained with the largest basis set (VQZ-F12). The latter procedure is based on the classification of the intermolecular excitation classes as detailed in [48,67]. The results are shown in Table 2. Beyond the total dispersion contributions, we also made use of the local analysis to separate the contribution of different molecular moieties in the PVE molecule (phenyl, ether oxygen and ethenyl). Shared orbitals are split up according to their NPA (natural population analysis) charges as described in [68].

**Table 2:** Comparison of different structures for PVE–MeOH dimers, with dispersion energies calculated at the LCCSD/VQZ-F12 level of theory (the parentheses contain the percentage of the fragment’s dispersion relative to the total dispersion energy).

	∆*E*_disp_(total) [kJ/mol]	∆*E*_disp_(phenyl) [kJ/mol]	∆*E*_disp_(O) [kJ/mol]	∆*E*_disp_(ethenyl) [kJ/mol]

OH–O	−14.3	−6.2 (43.2)	−5.1 (35.8)	−3.0 (21.1)
OH–O’	−15.7	−6.1 (38.7)	−4.9 (31.5)	−4.7 (29.8)
OH–P	−16.9	−11.9 (70.4)	−1.5 (9.2)	−3.5 (20.5)
OH–P’	−16.0	−14.0 (87.6)	−1.3 (8.0)	−0.7 (4.4)
OH–E	−15.6	−6.9 (44.3)	−2.1 (13.3)	−6.6 (42.4)
OH–E’	−13.1	−4.5 (34.0)	−0.9 (6.7)	−7.8 (59.3)

**Figure 2:**
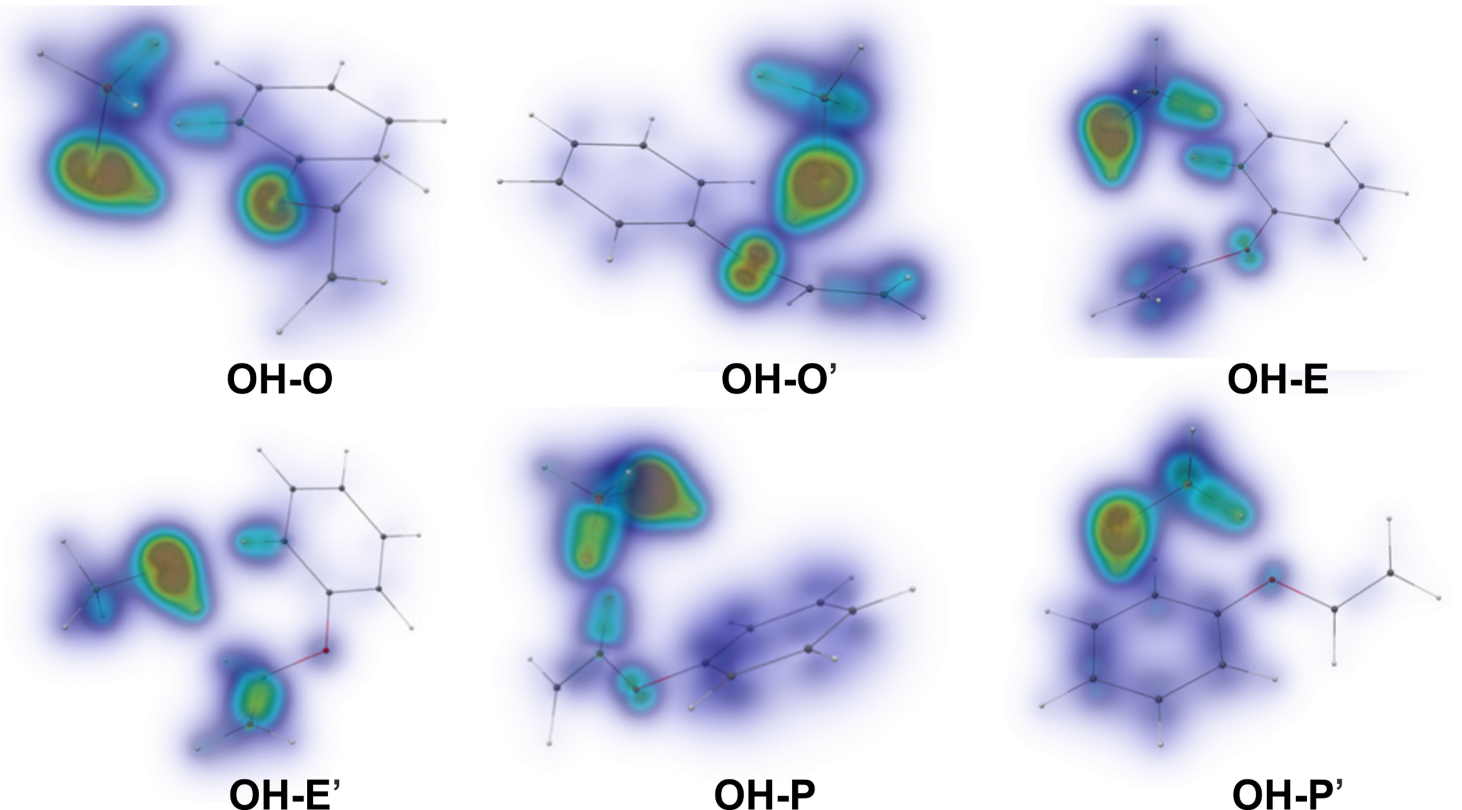
Dispersion interaction density (DID) plots calculated at the LCCSD/VQZ-F12 level. The brown zones indicate regions of electron density in a monomer which interact strongly by dispersion interactions with the other molecule. Blue stands for weaker/diffuse contributions. For example, in the top left figure one can observe that the OH group of methanol interacts strongly with the ether oxygen, with some dispersion energy coming as well from a CH orbital in the phenyl close to the methanol.

The dispersion interaction energies show an interesting pattern. Although all structures are significantly stabilized by dispersion, with a maximum energy difference of 2.6 kJ/mol when summed all together, the relative weight of the different molecular fragments varies quite significantly. The moiety with the largest potential as dispersion energy donor (DED) is the phenyl ring. This results in the strongest stabilization for the two conformers whereby the methanol is closest to the ring (OH–P and OH–P’). The other conformers have much more spread out contributions. What is surprising is that even for the ethenyl binding complexes the contribution of the phenyl ring is sizeable. Geometrically, this seems unlikely, given that the methanol moiety is not oriented favorably relative to the ring. The effect can, however, be understood by inspecting the respective dispersion interaction densities (DIDs, cf. Figure 2), which allow for an even finer-grained analysis. There, one can observe that the major contributor is not the π-system of the phenyl ring, but a C–H contact to the methanol (a similar effect had already been observed in diphenyl ether–methanol complexes [19]). This contact is reminiscent of stabilization effects observed in coupled diamondoids [69] or supramolecular complexes [70], where such interactions can be found in large numbers.

### Electronic ground state spectra

#### FTIR spectroscopy

**Figure 3:**
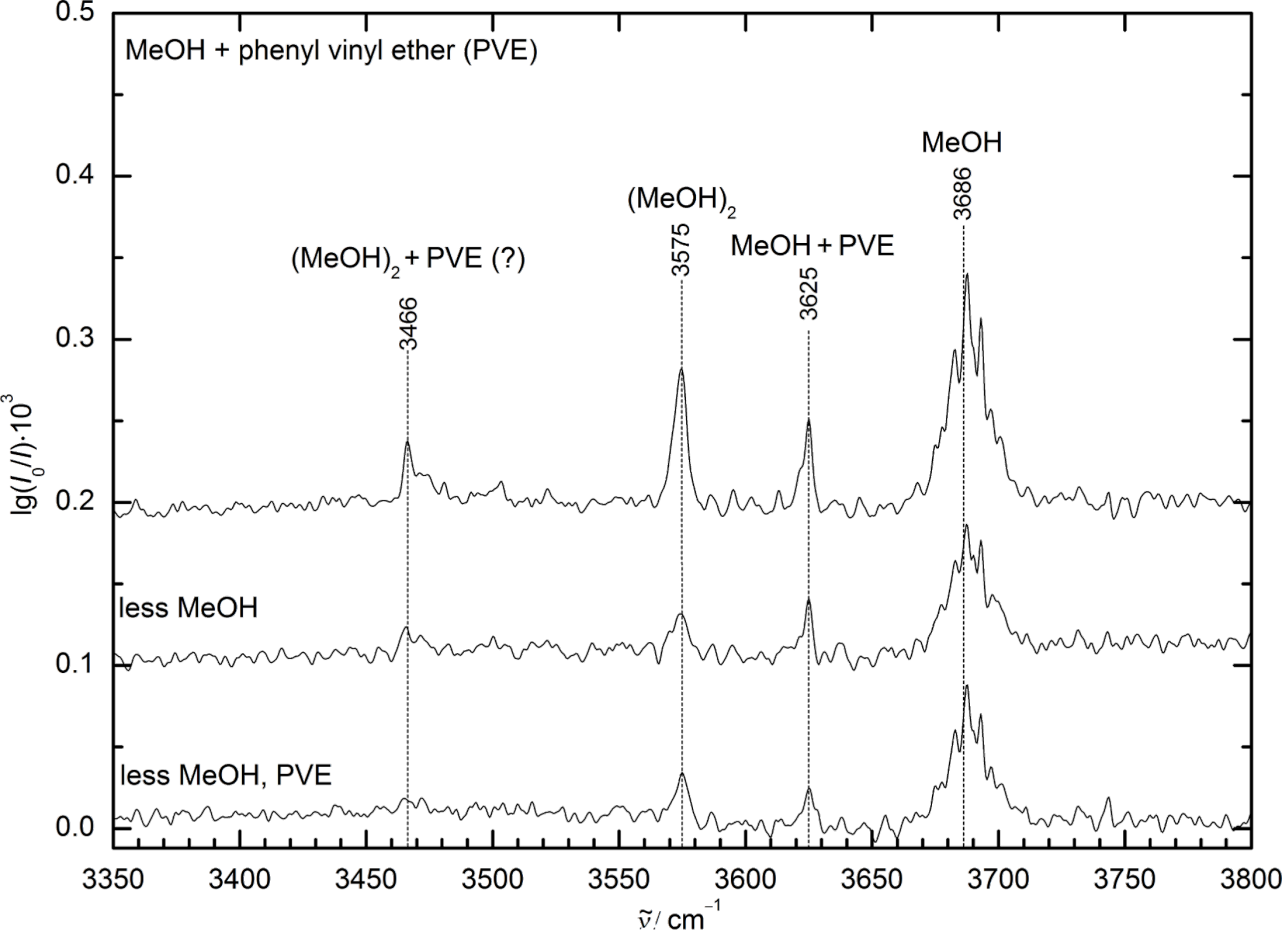
FTIR spectra of the supersonic expansion of methanol (MeOH) and phenyl vinyl ether (PVE) at different concentrations in helium. The spectra are spread out along the ordinate to improve visualization. Only one dominant mixed dimer band is visible in the spectra, lying at 3625 cm^−1^ (marked MeOH + PVE) between the methanol monomer at 3686 cm^−1^ (MeOH) and methanol dimer at 3575 cm^−1^ ((MeOH)_2_). By comparing the spectrum at the top with the other two spectra recorded at reduced concentrations of methanol (middle) or methanol and PVE (bottom), the further downshifted band at 3466 cm^−1^ can be attributed to a higher cluster, probably a methanol-rich mixed trimer ((MeOH)_2_ + PVE (?)), due to its scaling with the variation of the concentrations.

The results of an FTIR exploration of the conformational diversity of this system are shown in Figure 3. Besides methanol monomer, methanol dimer and a signal clearly attributed to a larger cluster, only a single, reasonably narrow absorption at 3625 cm^−1^ is observed. It can be attributed to mixed dimers of MeOH with PVE and allows for a single rigorous conclusion, due to the linearity of the technique and the comparable IR absorption cross section of all predicted dimer conformations (cf. Table 1 and Table S1, Supporting Information File 1): the global minimum structure and any other, higher lying isomers which are initially formed and impeded from relaxation to the global minimum due to broad or high interconversion barriers must have their OH stretching fundamental at 3625 ± 5 cm^−1^ or be significantly less abundant.

If one were to trust the relative harmonic wavenumber predictions from the preceding subsection (cf. Table 1), this would imply a single docking motif, as different docking motifs are predicted to lead to larger spectral separations. However, different extents of anharmonicity do not allow to completely ruling out overlapping docking motifs. Therefore, conformationally selective methods are desirable to investigate this possibility. Finally, the actual docking site has to be identified by structural or electronic excitation spectroscopy.

#### IR/R2PI spectroscopy

**Figure 4:**
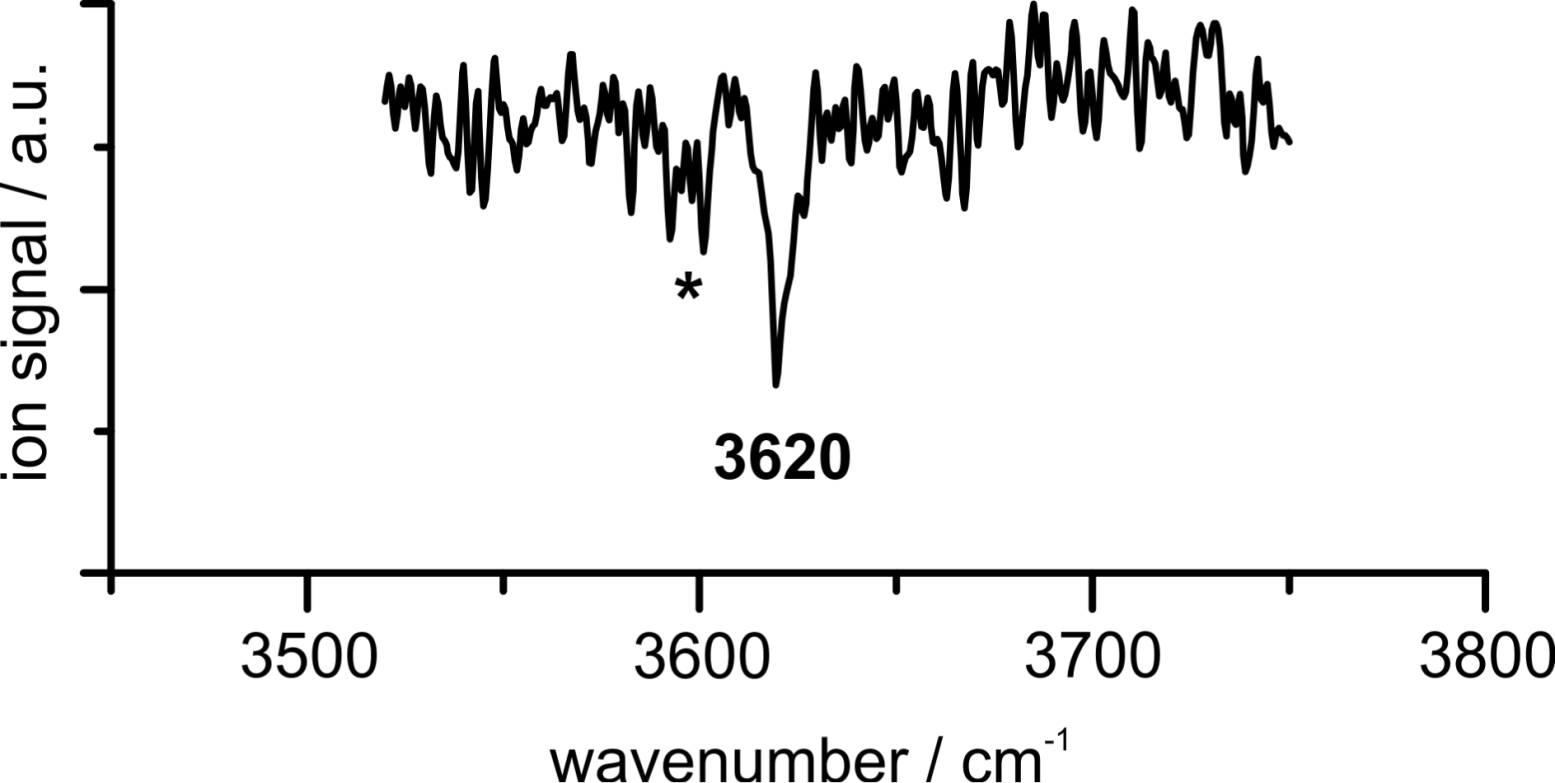
The IR/R2PI spectrum in the range of 3520–3750 cm^−1^ was obtained via the excitation energy of 36885 cm^−1^ using the carrier gas neon; the asterisk (*) indicates ionization-induced fragmentation from larger clusters (cf*.* Figure S2 in Supporting Information File 1).

Additional insight can be gained by using the mass- and isomer-selective IR/R2PI technique. This method requires knowledge on electronic excitation energies of the PVE–MeOH complex. For this reason, one-color R2PI spectra were recorded in the range of 36100–37600 cm^−1^ (cf. Figure S1 in Supporting Information File 1). While the R2PI spectrum of the PVE monomer shows well-resolved vibrational progressions (cf*.* Figure S1a, Supporting Information File 1), the spectrum of the solvent aggregate is broadened and affected by ionization-induced fragmentation of larger clusters (cf*.* Figure S1b, Supporting Information File 1). This is also reflected in the recorded IR/R2PI spectra (cf*.* Figure S2, Supporting Information File 1), yielding solely the spectrum shown in Figure 4 via the excitation energy of 36885 cm^−1^ containing an OH stretching vibration of a PVE–MeOH dimer.

Comparing the calculated OH stretching frequencies for the different isomers obtained at the DFT-D3 and SCS-CC2 levels (cf*.* Table 1) to the experiment does not allow for a clear structural assignment: the DFT-D3 calculations show the best agreement for the OH–P structure (3619 cm^−1^, scaling factor 0.9600). Regarding the relative electronic energies, the OH–O structure is somewhat favored, with OH–O’ coming as a close second. The latter frequencies are 3597 and 3600 cm^−1^, respectively, at the same level of theory. On the other hand, the computed SCS-CC2 frequencies would provide a coincident assignment, as both O-docking isomers would have the closest fundamentals compared to the measured frequency (3619 and 3621 cm^−1^). The assignment, however, would be tentative at best with this information alone. The OH–E isomers on the other hand can be excluded due to their lower OH stretching frequencies as well as the energetic disadvantage at the LCCSD(T0)-F12/CBS[T:Q] level (cf*.* Table 1).

In order to elucidate this problem, the electronic excitation energies can serve as a further indication for the binding motif, as shown for DPE–alcohol clusters before [20–22]. Comparing the vertical excitation energies for the different isomers with the experimental excitation energy of 36885 cm^–1^ yields the best agreement for the OH–O or OH–O’ isomer, which also show a significantly blue-shifted S_1_←S_0_ transition compared to the PVE monomer (adiabatic excitation energies of 38291 and 38164 cm^−1^, respectively, compared to 38034 cm^−1^ for the PVE monomer, cf*.* Table S2, Supporting Information File 1), as observed experimentally. In contrast to that, a red-shifted S_1_←S_0_ transition compared to PVE is predicted for the OH–P isomer (37907 cm^−1^), which would coincide with the fragmentation-dominated region of the R2PI spectrum, where, however, only signatures of larger clusters could be identified. These considerations strengthen the arguments for the presence of an OH∙∙∙O structure laid before, on the basis of the computed coupled cluster energies and the SCS-CC2 fundamental stretch frequencies. Additional experimental insight will be gained from the UV/IR/UV spectrum of the S_1_ state as well as the microwave investigations in the following section.

#### Chirp pulse Fourier transform microwave (CP-FTMW) spectroscopy

From the broadband CP-FTMW spectra obtained with neon as a carrier gas, we assigned two PVE–MeOH complexes with significantly different intensities. Complex 1 is about ten times more intense than complex 2. The experimental rotational constants (Table 3) for the two isomers agree the best with the values calculated for the OH–O’ isomer (as also indicated in the FTIR and the IR–UV investigations, which are, however, unable to distinguish OH–O from OH–O’) and the OH–P isomer, respectively. The identification of the two complexes to the OH–O’ and the OH–P isomers is guided by the absolute and relative values of the B and C rotational constants. Generally, the rotational constants calculated at the SCS-CC2/def2-TZVP level of theory agree somewhat better with the experimental values than the B3LYP-D3(BJ)/def2-TZVP values (note that we compare experimental *B*_0_ rotational constants with theoretical *B*_e_ rotational constants here). For the OH–P complex, however, we find that the B3LYP-D3(BJ)/def2-TZVP level of theory provides a better prediction of the magnitudes of the dipole-moment components. Experimentally, we only observe a-type transitions for this complex, which points to rather low values for μ_b_ and μ_c_. SCS-CC2 calculations predict all three dipole-moment components to be of comparable magnitude. At the B3LYP-D3(BJ) level, μ_a_ is predicted to be significantly stronger than μ_b_ and μ_c_. This change in magnitude for the dipole-moment components for different levels of calculation is more often observed for weakly bound complexes because the exact arrangement of the two monomers with respect to each other can have a major influence on the dipole-moment components. Also note that in none of the spectroscopic experiments, we observe the OH–E isomer that is also predicted to be of relatively low energy (cf*.* Table 1).

**Table 3:** Experimental rotational constants of the two observed complexes, using neon as carrier gas, that are assigned to the OH–O’ and the OH–P isomers, respectively. The experimental rotational parameters for the OH–O’ isomer (called Exp 1) are the results of a fit to a rigid-rotor asymmetric Hamiltonian including solely the A lines of the internal rotation splitting. Rotational parameters of a global fit (XIAM) including both A and E levels due to internal rotation for the OH–O’ isomer are presented in the Table S9 of Supporting Information File 1.

	Complex 1 (OH–O’ isomer)		Complex 2 (OH–P isomer)	

	Exp 1	SCS-CC2/def2-TZVP	Exp 1	SCS-CC2/def2-TZVP

*A* [MHz]	1466.59120(26)	1501.94	1275.7623(49)	1297.89
*B* [MHz]	697.48965(11)	697.58	818.45271(73)	818.01
*C* [MHz]	572.109900(95)	589.94	640.2184(11)	646.81
∆_J_ [kHz]	0.72697(62)		0.070(14)	
∆_JK_ [kHz]	−0.6669(26)		2.19(10)	
∆_K_ [kHz]	5.6217(62)		–	
δ_J_ [kHz]	0.15121(11)		–	
δ_K_ [kHz]	2.5783(29)		–	
A state transition	213 (49/104/60)		20(20/0/0)	
Dipole moment (D) (μ_a_/μ_b_/μ_c_)		2.2/1.9/1.2		0.8/0.4/0.8
σ [kHz]	6.7		7.9	

**Figure 5:**
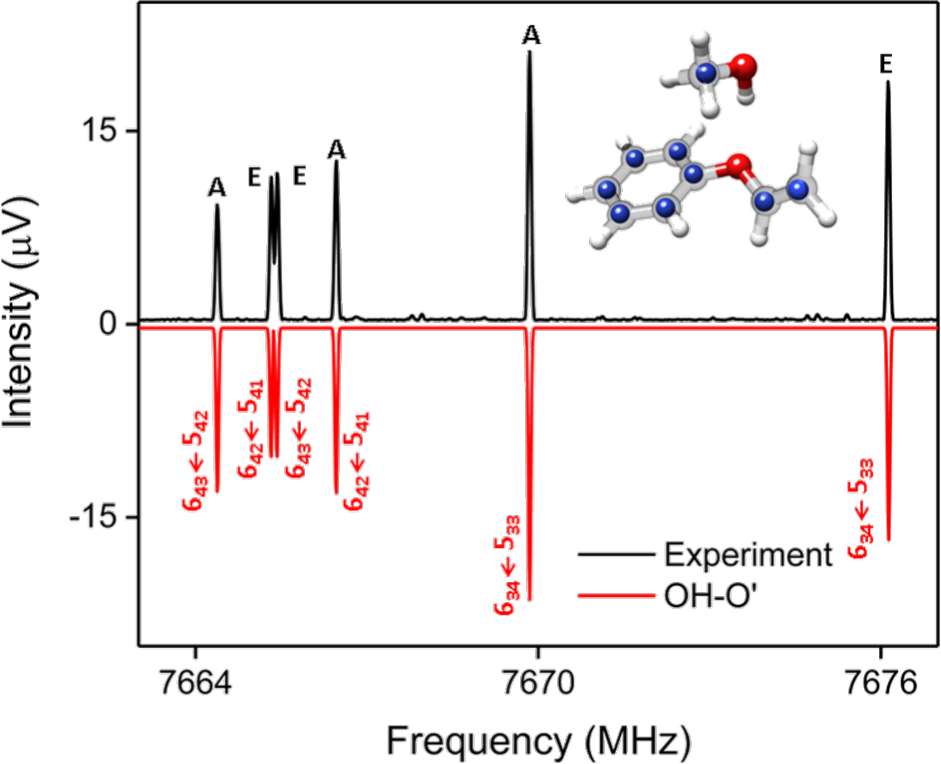
A section of the experimental 2–8 GHz spectrum using a mixture of PVE and MeOH (3 million acquisitions). The upper experimental trace in black is compared with simulations, based on fitted parameters that can be assigned to the OH–O’ isomer (complex 1, red) for the PVE–MeOHcomplex. The observed complex has a clear splitting pattern due to the internal rotation of the methyl group of methanol, labeled with A and E. The experimental ^13^C positions (blue atoms) (r_s_ substitution structure) deduced from a Kraitchman analysis are compared to the calculated structure at the SCS-CC2/def2-TZVP level of theory and further confirm the observation of the OH–O’ isomer.

The rotational spectra of the two isomers are qualitatively different. For the OH–O’ isomer (complex 1), we observe a characteristic line splitting into so-called A and E components (cf. Figure 5) arising from internal rotation of the methyl group of methanol, similar to the case of the DPE–MeOH complex. For the OH–P isomer (complex 2), no line splitting due to internal rotation was observed. This is consistent with the higher barrier for this motion due to the secondary interactions of the methyl group with PVE (cf. Figure 2).

Two different ways of analyzing the rotational spectrum of the OH–O’ isomer (complex 1) were performed. In Table 3, the results from a fit to an asymmetric-top Hamiltonian of only the A state species of the internal rotation splitting pair, which is often a good approximation, is summarized (Exp 1). In addition, we used the program XIAM to perform a global fit including both A and E lines. This global fit does not only provide the rotational constants, but also parameters of the internal rotor, in this case the methyl group. This includes the barrier height for internal rotation as well as the geometrical arrangement of the rotor with respect to the overall rotating molecule, as also discussed for the DPE–MeOH complex [19]. For PVE–MeOH, the barrier height was determined to be 261 cm^−1^, as summarized in Table S9 of Supporting Information File 1. This value is in agreement to barrier heights observed for other complexes with methanol [19]. It is somewhat lower than in the case of free methanol (373 cm^−1^) and also lower than the calculated barrier height of 341 cm^−1^ (cf*.* Table S9, Supporting Information File 1). This somewhat lower methyl group internal rotation barrier for the OH–O’ isomer could point to a softening of the C–O bond of methanol due to the hydrogen bond. The DID plots in Figure 2 also indicate that the methyl group is basically free from other interactions, so that no additional hindering is expected.

Furthermore, the transition intensities for the OH–O’ isomer are strong enough (with a signal-to-noise (SNR) of about 500:1 to 600:1 for the stronger transitions) to assign rotational transitions arising from all nine singly substituted ^13^C isotopologues in natural abundance (about 1%, cf*.* Figure S3, Supporting Information File 1). The additional data sets of rotational constants are summarized in Supporting Information File 1 (Table S12) together with line lists of the main isotopologues (Tables S10–S11) and the ^13^C isotopologues (Tables S13–S21). They allow us, using Kraitchman’s equations, to determine the carbon substitution structure, *r*_s_, of the complexes, which are the positions of the respective substituted carbon atoms with respect to the center of mass of the complex and thus the carbon backbone structure. The obtained *r*_s_ structure for complex 1 (cf*.* Figure 5) further confirms the assignment of complex 1 as the OH–O’ isomer, where the methyl group of the methanol moiety points towards the phenyl ring.

As mentioned, the OH–O’ isomer is about ten times more intense than the OH–P isomer. The intensity observed in CP-FTMW spectroscopy directly depends on the number of molecules, i.e., the population of the respective isomers, as well as the square of the transition dipole moments. Since the μ_a_ values for the two isomers differ by a factor of two (cf*.* Table S11 in Supporting Information File 1), the OH–P isomer can be considered to be about 2.5 times less populated than the OH–O’ isomer, as an upper estimate. Taking the predicted energy difference of 0.8 kJ/mol for granted, this ratio would correspond to a plausible [18] conformational freezing temperature of 100 K. A three-fold lower or three-fold higher conformational temperature appears unlikely, and thus a tentative experimental energy penalty for OH–P relative to OH–O’ ranges from 0.3 to 2 kJ/mol. This contradicts both inexpensive approaches (B3LYP-D3 and SCS-CC2 with def2-TZVP) and suggests that these methods somewhat underestimate the stability of OH∙∙∙O contacts.

### Electronically excited state spectrum

**Figure 6:**
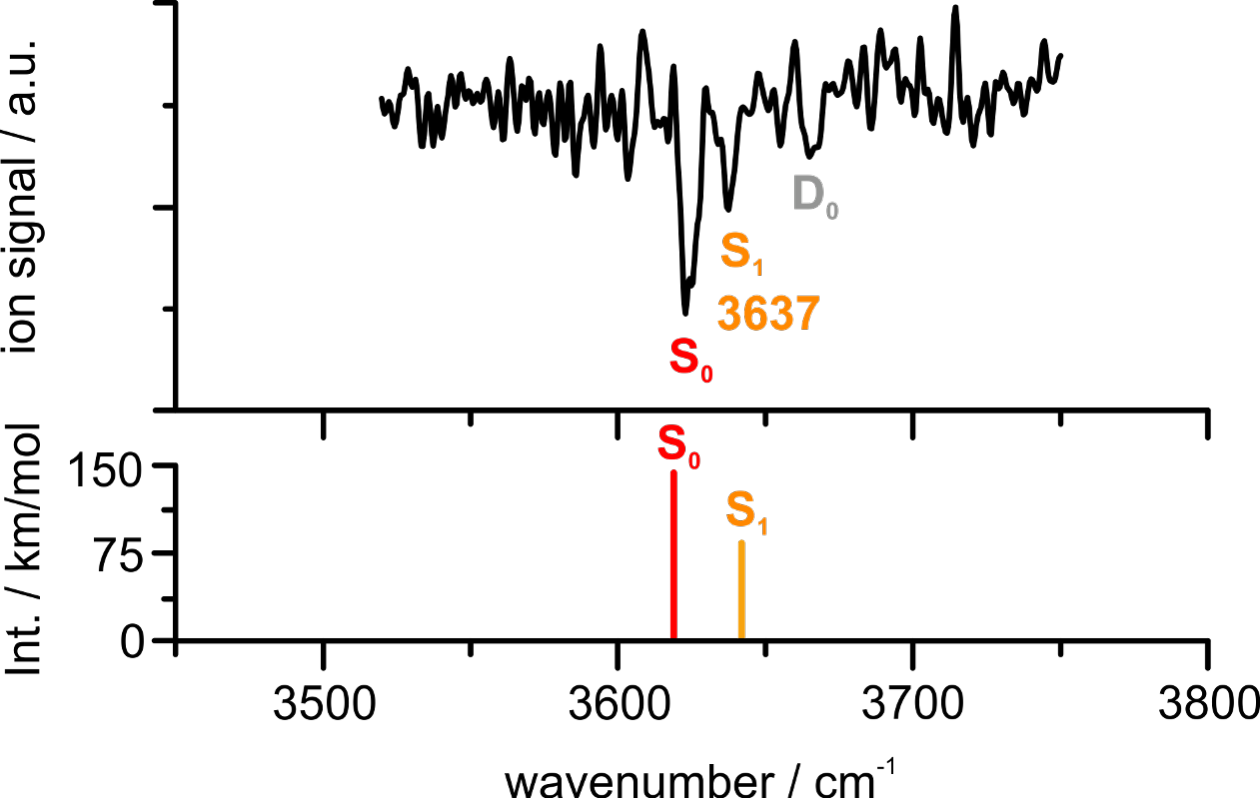
UV/IR/UV spectrum of PVE–MeOH in the range of 3520–3750 cm^−1^; excitation laser: 36741 cm^−1^, ionizing laser: 31847 cm^−1^, carrier gas helium; the lower trace shows the calculated OH stretching frequencies at the SCS-CC2/def2-TZVP level for the optimized S_0_ and S_1_ structure of the OH–O’ isomer scaled by 0.9635.

For the investigation of the electronically excited state by using the UV/IR/UV technique, a two-color R2PI signal is required. For this reason, the one-color R2PI signal was suppressed by attenuating the laser power of the excitation laser. On the other hand, higher pulse energies were used for the ionizing laser. The latter was set to 31847 cm^−1^ for the UV/IR/UV experiment in order to yield the best two-color R2PI signal. Figure 6 shows the recorded UV/IR/UV spectrum for the PVE–MeOH mass trace.

Due to temporally overlapping laser pulses, the spectrum contains transitions from the S_0_ state, the electronically excited (S_1_, at 3637 cm^−1^) and also the ionic D_0_ state (at 3667 cm^−1^). This could not be avoided, as the lifetime of the excited state, which is estimated to be in the order of 5–7 ns, is shorter than the laser pulse-widths of 7–10 ns. The OH stretching vibration at 3637 cm^−1^ originating from the electronically excited state of the PVE–MeOH complex is blue-shifted compared to the ground state, which indicates a decrease of the hydrogen bond strength in the S_1_ compared to the S_0_ state. A comparison with SCS-CC2 calculations shows a good agreement of a blue-shifted OH stretching frequency at 3642 cm^−1^ (cf*.* Table S2, Supporting Information File 1, scaled by 0.9635) compared to the ground state at 3619 cm^−1^ (cf*.* Table 1) for the OH–O’ isomer, which is also reflected in an increase of the H∙∙∙O hydrogen bond distance from 2.068 to 2.168 Å from S_0_ to S_1_ state geometry. This destabilization of the OH∙∙∙O hydrogen bond is further reflected in the calculated binding energies of the PVE–MeOH complex obtained at the SCS-CC2/def2-TZVP level, which are reduced by 0.9 kJ/mol regarding *D*_0_ and 1.6 kJ/mol regarding *D*_e_ in the S_1_ state compared to the S_0_ state for OH–O’ (cf*.* Table S3, Supporting Information File 1). The spectral shift can be explained by regarding the HOMO and LUMO orbitals involved in the S_1_←S_0_ transition, which is predicted to be mainly a π–π* transition with a small charge transfer contribution from the ether oxygen to the phenyl ring. The latter leads to a slightly decreased electron density at the binding site for the methanol molecule and therefore weakens the hydrogen bond. These findings are in line with observations in previous studies on diphenyl ether–alcohol complexes [20–21].

In principle, as the OH–O’ isomer has been identified in the S_0_ state, the observation of a respective OH∙∙∙O-bound structure can be expected in the S_1_ state as well. However, the OH–P isomers are predicted to be significantly stabilized in the S_1_ state (cf*.* Table S2, Supporting Information File 1). Nevertheless, due to the predicted red-shifts of the OH stretching frequencies of the OH–P isomers (indicating an increased hydrogen bond strength compared to the S_0_ state), their presence, i.e., by a rearrangement reaction from the OH–O’ isomer, can be excluded. By exciting the electronic origin of the OH–O’ isomer the formation of OH–E isomers can also be excluded as their expected excitation energies are higher than the one for OH–O’ and in addition they are energetically less stable (cf*.* Table S2, Supporting Information File 1).

## Conclusion

In this paper, the first spectroscopic and theoretical investigation on the isolated phenyl vinyl ether–methanol complex is presented. From the FTIR spectra, the existence of one isomer is concluded, which is confirmed by IR/UV spectroscopy in the electronic ground state (S_0_). The combined vibrational and electronic spectroscopic investigations, including a comparison of vibrational frequencies and electronic excitation energies, allow for an assignment of an OH∙∙∙O-bound structure. Broadband rotational (CP-FTMW) spectroscopy ultimately identifies OH–O’ as the observed isomer, ruling out the presence of the nearly isoenergetic OH–O. One explanation for its elusiveness would be a low interconversion barrier. However, rotational spectroscopy further reveals the presence of the OH–P isomer as a second isomer, being less populated, which is not observed with the less sensitive FTIR technique and might be superimposed by fragmentation of larger clusters in the usually more sensitive IR/UV experiments or it is even not populated due to different expansion conditions. No evidence was found for an OH∙∙∙ethenyl-bound structure, which is in agreement with the more pronounced energetic discrimination of OH–E isomers compared to the other binding motifs predicted at the LCCSD(T0)-F12/CBS[T:Q] level of theory.

In the electronically excited state (S_1_), the OH stretching vibration of the attached methanol undergoes a blue-shift compared to the S_0_ state. This indicates a weakening of the OH∙∙∙O bond upon electronic excitation compared to the ground state and is in good agreement with the calculated frequency shift for the S_0_ and S_1_ state structures obtained at the SCS-CC2/def2-TZVP level and is furthermore in line with findings for similar diphenyl ether–alcohol complexes from previous investigations [20–21].

In summary, we present a multi-spectroscopic analysis on a molecular complex with a very delicate balance between, for the first time, three different binding motifs. This provides an excellent benchmark system for theory, since DFT-D3 as well as SCS-CC2 methods fail in predicting the correct energetic order, whereas LCCSD(T0)-F12 succeeds in the preferred docking motif. These differences are in the range of only 2 kJ/mol, when considering relative electronic energies, but that is already enough to tip the scales in the wrong direction. Comparing VTZ-F12 and VQZ-F12 results, we observe that the electronic energies are well converged for the smaller basis (Table S6, Supporting Information File 1). This would place the main accuracy bottleneck in the electronic structure method (i.e., functional, correlation truncation) chosen.

Finally, regarding the docking preference in comparison to the previously investigated diphenyl ether complex with methanol, a conclusion might be that methanol needs the interaction with a second phenyl ring in order to prefer the OH∙∙∙π motif over OH∙∙∙O, as observed for diphenyl ether. The secondary interaction of methanol with a smaller ethenyl moiety being present in phenyl vinyl ether instead of a phenyl ring seems to be insufficient to favor the phenyl docking site.

## Supporting Information

File 1 Additional computational and experimental data.
