# Supplementary material for: The phenyl vinyl ether–methanol complex: a model system for quantum chemistry benchmarking
Source: Beilstein J Org Chem. 2018 Jul 2;14:1642–54. doi: 10.3762/bjoc.14.140 (PMC6036964; doi:10.3762/bjoc.14.140)
Supplement: File 1 — Additional computational and experimental data. [file Beilstein_J_Org_Chem-14-1642-s001.pdf]

Supporting Information

for

The phenyl vinyl ether–methanol complex: a model system for quantum chemistry benchmarking

Dominic Bernhard[1], Fabian Dietrich[1], Mariyam Fatima[2,3], Cristóbal Pérez[2,3], Hannes C. Gottschalk[5], Axel Wuttke[5], Ricardo A. Mata[5]*, Martin A. Suhm[5]*, Melanie Schnell[2,3,4]* and Markus Gerhards[1]*

Address: [1]Fachbereich Chemie & Research Center Optimas, Technische Universität Kaiserslautern, Erwin-Schrödinger-Str. 52, D-67663 Kaiserslautern, Germany, [2]Max Planck Institute for the Structure and Dynamics of Matter, Luruper Chaussee 149, D-22761 Hamburg, Germany, [3]Deutsches Elektronen Synchrotron (DESY), Notkestrasse 85, D-22607 Hamburg, Germany, [4]Institute of Physical Chemistry, Christian-Albrechts-Universität zu Kiel, Max-Eyth-Strasse 1, D-24118 Kiel, Germany and [5]Institut für Physikalische Chemie, Georg-August-Universität Göttingen, Tammannstrasse 6, D-37077 Göttingen, Germany

Email: Ricardo A. Mata – rmata@gwdg.de; Martin A. Suhm – msuhm@gwdg.de; Melanie Schnell – melanie.schnell@desy.de; Markus Gerhards – gerhards@chemie.uni-kl.de

* Corresponding author

Additional computational and experimental data

Table S1: Comparison of different structures for PVE–MeOH dimers in the S_0 with electronic energies E_{rel} and zero-point corrected energies $E_{0,\mathrm{rel}}$ relative to the OH-O isomer, for the level B3LYP-D3(BJ)/def2-TZVP and SCS-CC2/def2-TZVP.

	B3LYP-D3		SCS-CC2	
	E_{rel} / kJ/mol	$E_{0,\mathrm{rel}}$ / kJ/mol	E_{rel} / kJ/mol	$E_{0,\mathrm{rel}}$ / kJ/mol
OH–O	0.0	0.0	0.0	0.0
OH–O'	-0.1	0.0	-0.3	-0.5
OH–P	0.4	-0.1	-1.1	-2.1
OH–P'	3.2	1.5	2.7	1.0
OH–E	-0.3	-0.3	0.7	0.5
OH–E'	2.8	2.4	4.2	3.8

Table S2: Comparison of different structures for PVE–MeOH dimers in the S_1 with electronic energies E_{rel} and zero-point corrected energies $E_{0,\mathrm{rel}}$ relative to the OH–O isomer, adiabatic excitation wavenumber $\tilde{\nu}_{adia}$, the scaled wavenumbers $\tilde{\nu}$ of the OH-stretching vibration together with the respective IR intensity I as well as the difference of the wavenumbers $\tilde{\nu}$ between ground and excited state, at the SCS-CC2/def2-TZVP level (scaling factor: 0.9635).

	E_{rel} / kJ/mol	$E_{0,\mathrm{rel}}$ / kJ/mol	$\tilde{\nu}$ / cm^{-1}	I / km/mol	$\Delta\tilde{\nu}$ / cm^{-1}	$\tilde{\nu}_{adia}$/ cm^{-1}
OH–O	0.0	0.0	3638	173	+19	38291
OH–O'	-1.8	-2.4	3642	83	+21	38164
OH–P	-5.8	-7.1	3567	128	-64	37907
OH–P'	-2.4	-3.7	3594	124	-42	37875
OH–E	1.8	0.3	3631	66	+24	38303
OH–E'	4.6	3.9	3633	66	+27	38331
PVE monomer						38034

Table S3: Comparison of the dissociation energies of the different structures for PVE–MeOH dimers in the S_0 and S_1 without (D_e) and with (D_0) zero-point correction, calculated at the SCS-CC2/def2-TZVP level.

	S_0		S_1	
	D_e / kJ/mol	D_0 / kJ/mol	D_e / kJ/mol	D_0 / kJ/mol
OH–O	-20.6	-16.4	-17.5	-13.6
OH–O'	-20.9	-16.9	-19.3	-16.0
OH–P	-21.7	-18.5	-23.3	-20.7
OH–P'	-17.9	-15.7	-20.0	-17.3
OH–E	-19.9	-15.9	-16.8	-13.3
OH–E'	-16.5	-12.6	-13.0	-9.7

Table S4: F12b corrected triples contributions to E_{rel} @ LLCSD(T0)-F12b/VQZ-F12 for different structures of PVE–MeOH dimers.

	(T0)-F12b E_{rel}(T0) / kJ/mol
OH–O	0.0
OH–O'	-0.2
OH–P	-0.8
OH–P'	-0.4
OH–E	-0.5
OH–E'	0.0

Table S5: Comparison of LCCSD(T0)-F12b results @ VTZ-F12 and VQZ-F12 basis sets for different structures of PVE–MeOH dimers.

	E_{rel} / kJ/mol VTZ-F12	E_{disp} / kJ/mol VTZ-F12	E_{rel} / kJ/mol VQZ-F12	E_{disp} / kJ/mol VQZ-F12
OH–O	0.0	-13.7	0.0	-14.3
OH–O'	0.3	-15.1	0.2	-15.7
OH–P	1.3	-16.4	1.1	-16.9
OH–P'	2.4	-15.3	2.4	-16.0
OH–E	2.2	-15.2	2.1	-15.6
OH–E'	4.7	-12.7	4.7	-13.1

Table S6: Energy decomposition analysis for different structures of PVE–MeOH dimers @LCCSD/VQZ-F12.

	E_{disp} / kJ/mol VQZ-F12	E_{exdisp} / kJ/mol VQZ-F12	E_{ionic} / kJ/mol VQZ-F12
OH–O	-14.3	0.1	-8.3
OH–O'	-15.7	0.1	-8.4
OH–P	-16.9	0.1	-9.5
OH–P'	-16.0	0.1	-7.8
OH–E	-15.6	0.1	-10.9
OH–E'	-13.1	0.1	-9.6

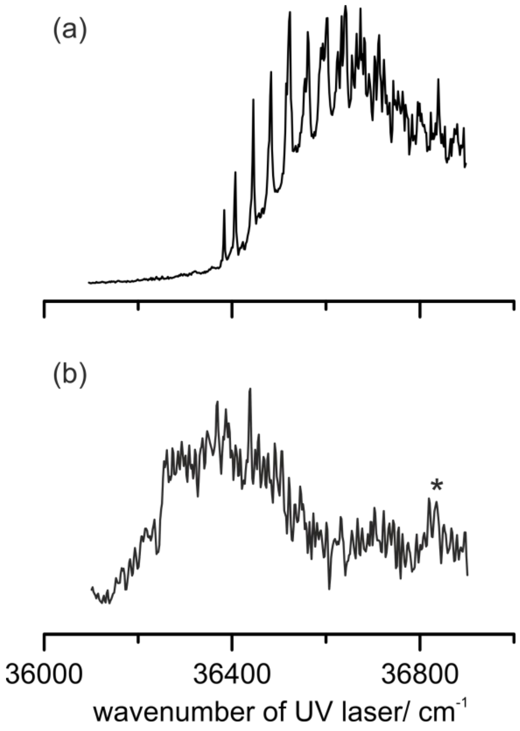

Figure S1: R2PI spectra obtained with the carrier gas neon are shown. The PVE monomer spectrum (a) exhibits a pronounced vibrational progression, which is absent in the spectrum of the PVE–MeOH dimer (b). Trace (b) shows the R2PI spectrum of the PVE–MeOH mass trace with a sufficiently high MeOH partial pressure for applying the IR/R2PI technique with an adequate ion signal at 36885 cm^{-1} (marked with *). The broad transition around 36400 cm^{-1} is a contamination due to fragmentation of larger clusters.

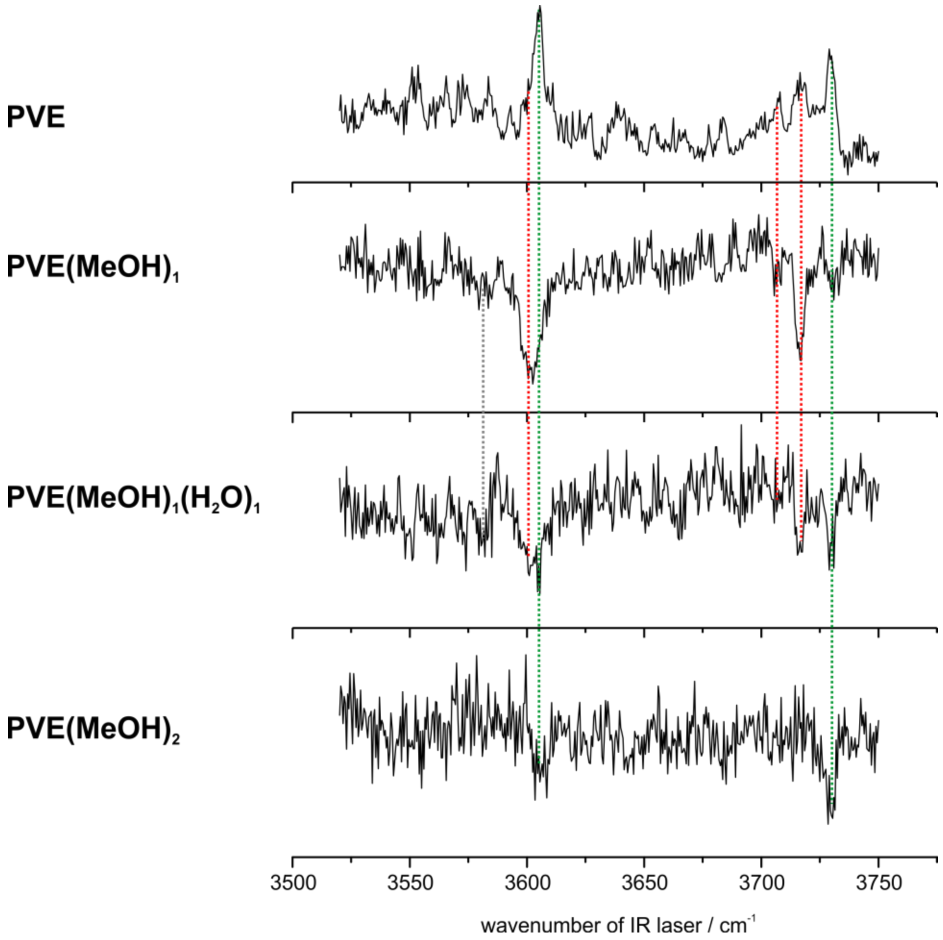

Figure S2: IR/R2PI spectra for different mass traces, dashed lines indicate fragmentation; UV excitation: 36359 cm^{-1}, carrier gas neon. Comparing the different mass traces leads to the conclusion that all vibrational transitions on the PVE–MeOH mass trace can be explained by fragmentation of larger clusters involving more than one methanol molecule and/or water molecules.

Synthesis of phenyl vinyl ether (PVE)

25 g of β-bromophenethol and 25 g of *t*-BuOK were dissolved in THF (100 mL) and heated under reflux and inert atmosphere for 2 h. Afterwards the mixture was cooled, filtrated and concentrated. The mixture was diluted with diethyl ether and washed with water. The organic layer was dried over $MgSO_4$. Finally, the colorless liquid was purified by distillation, yielding 3.01 g (20.1%).

^{1}H-NMR (400 MHz, $CDCl_3$) δ/ppm: 7.37 (dd, $^3J_{HH}$ = 8.5 Hz Hz, $^3J_{HH}$ = 7.5 Hz, 2H), 7.12 (t, $^3J_{HH}$ = 7.4 Hz, 1H), 7.05 (d, $^3J_{HH}$ = 8.6 Hz, 2H), 6.70 (dd, $^3J_{HH}$ = 13.7 Hz, $^3J_{HH}$ = 6.1 Hz, 1H), 4.82 (dd, $^3J_{HH}$ = 13.7 Hz, $^2J_{HH}$ = 1.5 Hz, 1H), 4.48 (dd, $^3J_{HH}$ = 6.1 Hz, $^2J_{HH}$ = 1.5 Hz, 1H)

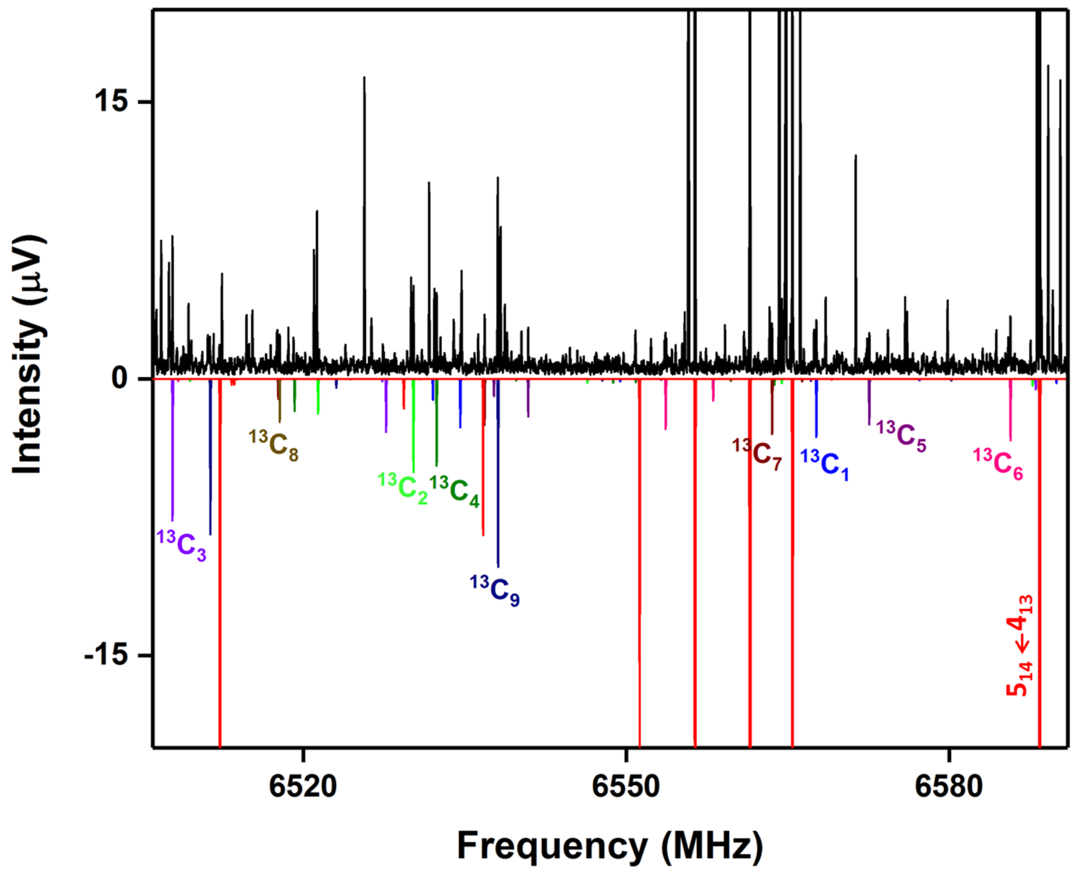

Figure S3: A section of the experimental 2–8 GHz rotational spectrum using a mixture of phenyl vinyl ether and methanol (3 million acquisitions). The upper experimental trace in black is compared with simulations based on fitted parameters for the main isotopologue of the OH–O' isomer (bottom trace, red) and its nine ^{13}C isotopologues (bottom trace, multiple colours).

Table S7: Calculated rotational constants and dipole moment components for the different PVE–MeOH isomers obtained at the B3LYP-D3(BJ)/def2-TZVP level.

	A / MHz	B / MHz	C / MHz	μ_a / D	μ_b / D	μ_c / D
OH-O	1505.30	698.06	503.98	1.7	1.8	1.7
OH-O'	1527.97	691.24	581.32	2.3	1.8	1.1
OH-P	1293.41	814.13	645.14	1.4	0.3	0.4
OH-P'	1364.17	751.30	599.39	1.4	1.8	0.5
OH-E	1585.23	682.04	538.05	0.8	0.7	0.3
OH-E'	1690.10	608.61	462.98	0.2	0.2	1.6

Table S8: Calculated rotational constants and dipole moment components for the different PVE–MeOH isomers obtained at the SCS-CC2/def2-TZVP level.

	A / MHz	*B* / MHz	*C* / MHz	μ_a / D	μ_b / D	μ_c / D
OH-O	1450.34	707.31	505.77	1.5	1.9	1.8
OH-O'	1501.94	697.58	589.94	2.2	1.9	1.1
OH-P	1297.89	818.01	646.81	0.8	0.4	0.8
OH-P‘	1332.08	771.57	606.41	1.2	0.9	0.9
OH-E	1573.98	677.14	536.24	0.7	0.7	0.3
OH-E'	1692.22	585.85	448.56	0.5	0.2	1.5

Table S9: Experimental rotational constants of the two experimentally observed complexes (using neon

	Complex 1 (OH–O' isomer)			Complex 2 (OH–P isomer)	
	Exp 1	XIAM	SCS-CC2/def2-TZVP	Exp 1	SCS-CC2/def2-TZVP
A(MHz)	1466.59120(26)	1466.56596(21)	1501.94	1275.7611(15)	1297.89
B(MHz)	697.48965(11)	697.48146(15)	697.58	818.45753(37)	818.01
C (MHz)	572.109900(95)	572.083981(91)	589.94	640.22299(52)	646.81
Δ_J (kHz)	0.72697(62)	0.62736(72)		0.2052(83)	
Δ_{JK} (kHz)	-0.6669(26)	-0.0800(24)		1.992(43)	
Δ_K (kHz)	5.6217(62)	5.1315(45)		-	
δ_J (kHz)	0.15121(11)	-0.15072(13)		-	
δ_K (kHz)	2.5783(29)	-0.048134(87)		-	
F_0 (cm^{-1})	-	5.3		-	
V_3(cm^{-1})	-	261.838(58)	341	-	
D_{pi2J}(Hz)	-	0.18322(85)		-	
D_{pi2K}(Hz)	-	0.5968(19)		-	
D_{pi2-}(Hz)	-	0.22996(60)		-	
δ(rad)	-	1.23622(30)	2.8	-	
ε(rad)	-	4.4729(40)	0.2	-	
A state transition	213 (49/104/60)	211		20(20/0/0)	
E state transition	-	140		-	
Dipole Moment (D) (μ_a/μ_b/μ_c)			2.2/1.9/1.2		0.8/0.4/0.8
σ (kHz)	6.68	6.18		5.55	

as carrier gas) that can be assigned to the OH-O' and the OH-P isomers.

Table S10: Line list of A and E state fit (using XIAM) for complex 1 (OH–O' isomer) for PVE–MeOH.

State	J'	K_a'	K_c'		J''	K_a''	K_c''	ν_{obs}	ν_{obs}-ν_{calc}
A	1	1	1	←	0	0	0	2038.6993	-0.0002
E								2038.4288	-0.0016
A	5	2	3	←	5	1	4	2067.1185	-0.0012
E								2067.9416	-0.0001
A	4	2	2	←	4	1	3	2099.1239	0.0007
A	5	1	4	←	5	0	5	2099.9154	-0.0083
E								2104.5901	-0.0089
A	6	2	4	←	6	1	5	2137.5374	-0.0001
E								2138.4090	-0.0002
A	1	1	0	←	0	0	0	2164.0540	-0.0137
E								2164.5137	-0.0045
A	3	2	1	←	3	1	2	2197.9915	0.0020
E								2200.8927	0.0048
A	7	2	5	←	7	1	6	2337.0128	-0.0044
E								2338.3061	-0.0009
A	2	1	2	←	1	1	1	2413.8168	0.0036
E								2414.2624	0.0021
A	2	0	2	←	1	0	1	2525.0671	0.0013
E								2524.8262	-0.0061
A	2	1	1	←	1	1	0	2664.5422	-0.0001
E								2664.2432	0.0029
A	8	2	6	←	8	1	7	2684.0281	0.0037
E								2686.3565	0.0063
A	6	1	5	←	6	0	6	2705.4140	-0.0069
E								2716.1131	-0.0067
A	3	0	3	←	2	1	1	2719.4974	0.0106
A	8	3	6	←	8	2	6	2859.2036	0.0004
A	3	2	2	←	3	1	3	2879.7664	-0.0020
E								2876.5597	-0.0054
A	3	0	3	←	2	1	2	3095.5775	-0.0065
E								3094.5435	-0.0002
A	4	1	3	←	3	2	1	3099.5323	-0.0005
E								3096.4881	0.0050
A	4	2	3	←	4	1	4	3144.9321	-0.0057
E								3142.3029	-0.0097
A	9	3	6	←	9	2	7	3146.9450	-0.0005
E								3148.1128	0.0010
A	7	3	5	←	6	4	2	3162.1115	-0.0026
A	10	3	7	←	10	2	8	3162.5532	0.0076
E								3163.7656	0.0068
A	7	3	5	←	6	4	3	3164.7157	-0.0026
A	4	1	3	←	3	2	2	3168.9227	-0.0040
A	2	1	2	←	1	0	1	3182.9172	0.0007
E								3183.1838	-0.0007
A	9	2	7	←	9	1	8	3185.0938	0.0056
E								3189.7252	0.0144
A	7	3	5	←	7	2	5	3250.6348	-0.0023
E								3248.9948	-0.0065
A	6	2	5	←	5	3	2	3253.1371	0.0092
A	8	3	5	←	8	2	6	3254.4901	0.0009
E								3255.9773	-0.0039
A	11	6	6	←	10	7	4	3349.1749	-0.0004
A	11	6	6	←	10	7	3	3349.1749	0.0045
A	7	3	4	←	6	4	2	3353.8957	-0.0081
A	7	3	4	←	6	4	3	3356.5075	-0.0004
A	7	1	6	←	7	0	7	3407.6856	-0.0075
E								3429.4353	-0.0070
A	7	3	4	←	7	2	5	3442.4279	0.0011
E								3444.9393	-0.0028
A	5	2	4	←	5	1	5	3479.1762	-0.0077
E								3474.2922	-0.0067
A	4	2	3	←	4	0	4	3516.9938	-0.0118
E								3518.4562	-0.0079
A	6	3	4	←	6	2	4	3580.2035	0.0022
E								3575.4084	0.0060
A	3	1	3	←	2	1	2	3612.3211	0.0033
E								3612.8699	0.0016
A	19	1	18	←	18	4	15	3617.4946	-0.0075
A	6	3	3	←	6	2	4	3660.2611	0.0011
E								3665.5425	-0.0038
A	4	0	4	←	3	1	2	3678.8466	0.0178
E								3676.7032	0.0030
A	5	2	4	←	5	0	5	3726.2220	-0.0067
E								3731.2430	-0.0013
A	3	0	3	←	2	0	2	3753.4369	0.0022
E								3752.8972	0.0013
A	3	2	2	←	2	2	1	3808.7405	0.0055
E								3814.7452	0.0010
A	5	3	3	←	5	2	3	3832.8442	0.0020
A	5	3	2	←	5	2	3	3860.2306	0.0016
E								3871.5691	0.0028
A	3	2	1	←	2	2	0	3864.0212	0.0016

E								3857.9930	0.0029
A	6	2	5	←	6	1	6	3880.5276	-0.0098
E								3869.7346	-0.0084
A	20	1	19	←	19	3	16	3956.3996	0.0114
A	3	1	2	←	2	1	1	3987.3918	-0.0017
E								3987.2537	-0.0015
A	4	3	2	←	4	2	2	4002.6576	0.0011
A	4	3	1	←	4	2	2	4009.6053	0.0018
A	6	2	5	←	6	0	6	4033.8156	-0.0112
E								4045.6812	-0.0033
A	6	2	4	←	5	3	2	4062.2743	0.0147
A	6	2	4	←	5	3	3	4089.6452	-0.0011
A	9	4	6	←	8	5	3	4138.5471	-0.0089
A	3	3	1	←	3	2	2	4167.0002	-0.0004
E								4149.2759	0.0043
A	13	4	9	←	13	3	10	4181.7672	-0.0095
A	4	3	2	←	4	2	3	4203.4649	-0.0026
E								4186.6602	0.0029
A	4	3	1	←	4	2	3	4210.4130	-0.0015
E								4229.4672	0.0019
A	3	1	3	←	2	0	2	4270.1753	0.0068
E								4271.2274	0.0069
A	5	3	3	←	5	2	4	4273.6507	-0.0061
E								4262.7452	0.0062
A	5	3	2	←	5	2	4	4301.0422	-0.0014
E								4312.8575	-0.0051
A	7	2	6	←	6	3	3	4309.6326	0.0004
E								4305.2910	0.0022
A	7	2	6	←	7	1	7	4344.2213	-0.0115
E								4322.4081	-0.0089
A	6	3	4	←	6	2	5	4389.3269	-0.0061
E								4384.2424	-0.0045
A	7	2	6	←	6	3	4	4389.6875	-0.0033
E								4395.4310	-0.0018
A	8	3	6	←	7	4	3	4426.4382	-0.0053
A	4	0	4	←	3	1	3	4430.0025	0.0008
E								4427.1558	0.0009
A	7	2	6	←	7	0	7	4434.5097	-0.0128
E								4458.7385	-0.0086
A	6	3	3	←	6	2	5	4469.3881	-0.0035
A	5	1	4	←	4	2	2	4489.2727	0.0020
E								4487.8361	0.0040
A	7	3	5	←	7	2	6	4560.8219	-0.0029
E								4557.9984	-0.0051
A	5	1	4	←	4	2	3	4690.0769	-0.0048
E								4690.3759	-0.0030
A	7	3	4	←	7	2	6	4752.6156	0.0011
A	8	3	6	←	8	2	7	4795.9224	0.0014
E								4793.0288	0.0046
A	4	1	4	←	3	1	3	4802.0817	0.0123
E								4803.2968	-0.0095
A	8	3	5	←	7	4	3	4821.7091	-0.0204
A	8	3	5	←	7	4	4	4831.1476	-0.0011
A	8	2	7	←	8	1	8	4862.8807	-0.0017
E								4826.6968	-0.0084
A	8	2	7	←	8	0	8	4914.0457	-0.0055
A	9	1	8	←	9	1	9	4915.2637	0.0092
E								4855.9680	0.0055
A	9	1	8	←	9	0	9	4943.4384	0.0067
A	4	0	4	←	3	0	3	4946.7377	0.0022
E								4945.4815	0.0021
A	2	2	1	←	1	1	0	4971.7925	-0.0038
E								4962.8709	0.0091
A	2	2	0	←	1	1	0	4985.9090	0.0034
E								4994.3983	0.0051
A	3	1	2	←	2	0	2	5021.3606	0.0193
E								5021.6742	-0.0009
A	4	2	3	←	3	2	2	5067.2432	0.0044
E								5069.0542	0.0004
A	2	2	1	←	1	1	1	5097.1610	-0.0034
E								5088.9539	0.0044
A	9	3	7	←	9	2	8	5099.1006	0.0041
A	4	3	2	←	3	3	1	5103.7078	0.0021
E								5106.4411	0.0016
A	4	3	1	←	3	3	0	5109.6530	0.0000
E								5106.8722	0.0015
A	2	2	0	←	1	1	1	5111.2735	-0.0002
E								5120.4993	0.0183
A	8	1	8	←	7	2	6	5127.1700	0.0126
A	9	4	6	←	9	3	6	5143.0963	-0.0068
A	8	3	5	←	8	2	7	5191.2128	0.0058
A	4	2	2	←	3	2	1	5198.6522	-0.0038
E								5196.8190	-0.0042
A	9	4	5	←	9	3	6	5212.8921	-0.0104
A	8	2	7	←	7	3	4	5237.4218	-0.0034
E								5237.3534	-0.0089

A	4	1	3	←	3	1	2	5297.5219	-0.0004
E								5297.3709	-0.0001
A	4	1	4	←	3	0	3	5318.8082	0.0050
E								5321.6370	0.0061
A	8	4	5	←	8	3	5	5416.1532	-0.0015
A	9	2	8	←	9	1	9	5427.2718	-0.0070
A	8	2	7	←	7	3	5	5429.2118	-0.0031
E								5433.3040	0.0010
A	8	4	4	←	8	3	5	5443.8249	-0.0071
A	10	4	7	←	9	5	4	5457.8588	0.0120
A	8	3	6	←	8	1	7	5543.2210	-0.0066
A	7	3	5	←	7	1	6	5587.6496	-0.0047
E								5587.3043	-0.0040
A	7	4	4	←	7	3	4	5597.4823	-0.0011
E								5572.0947	-0.0022
A	7	4	3	←	7	3	4	5606.9025	-0.0002
E								5628.8903	-0.0076
A	9	3	7	←	9	1	8	5611.1135	-0.0073
E								5616.4872	-0.0017
A	10	4	6	←	9	5	5	5616.7348	-0.0101
A	7	2	5	←	6	3	3	5619.8165	-0.0035
E								5614.2934	0.0024
A	9	3	7	←	8	4	4	5651.2091	-0.0117
E								5633.4083	-0.0035
A	7	0	7	←	6	1	5	5672.9200	0.0128
E								5650.4977	0.0004
A	7	2	5	←	6	3	4	5699.8748	-0.0038
E								5704.4367	0.0017
A	6	4	3	←	6	3	3	5705.7384	-0.0004
E								5674.2222	0.0051
A	6	4	2	←	6	3	3	5708.3416	-0.0014
E								5730.5546	0.0053
A	10	1	9	←	10	0	10	5710.6359	0.0067
E								5767.6358	0.0089
A	6	3	4	←	6	1	5	5717.7433	0.0045
A	5	0	5	←	4	1	4	5735.1065	0.0106
E								5728.1121	0.0196
A	5	4	2	←	5	3	2	5763.8788	-0.0010
A	6	4	3	←	6	3	4	5785.7951	-0.0023
E								5764.3756	0.0145
A	6	4	2	←	6	3	4	5788.4011	-0.0005
E								5820.6996	0.0064
A	7	4	4	←	7	3	5	5789.2740	0.0009
E								5768.0377	0.0000
A	5	4	2	←	5	3	3	5791.2677	0.0012
E								5775.0638	-0.0039
A	5	4	1	←	5	3	3	5791.7935	0.0017
A	4	4	0	←	4	3	1	5791.9488	0.0070
E								5802.6331	0.0017
A	7	4	3	←	7	3	5	5798.6884	-0.0040
E								5824.8406	0.0020
A	4	4	1	←	4	3	2	5798.8265	-0.0037
E								5788.9139	-0.0035
A	10	3	8	←	10	1	9	5803.2045	0.0004
A	8	4	5	←	8	3	6	5811.4312	-0.0095
E								5795.4287	0.0053
A	8	4	4	←	8	3	6	5839.1182	0.0002
E								5857.5462	0.0060
A	9	4	6	←	9	3	7	5864.0152	-0.0008
E								5855.0251	0.0120
A	11	3	9	←	11	2	10	5908.9726	0.0114
A	10	4	7	←	10	3	8	5959.2958	0.0042
A	5	1	5	←	4	1	4	5982.1444	0.0037
E								5985.0407	0.0028
A	9	2	8	←	8	3	5	5995.9447	-0.0116
E								5999.7705	0.0017
A	10	2	9	←	10	1	10	6027.5474	-0.0042
E								5970.5881	0.0026
A	4	3	2	←	4	1	3	6101.7885	0.0089
A	8	0	8	←	7	1	6	6102.8191	0.0012
A	5	0	5	←	4	0	4	6107.1607	-0.0029
E								6104.2476	0.0036
A	3	2	2	←	2	1	1	6115.9925	0.0034
E								6113.3669	0.0012
A	3	2	1	←	2	1	1	6185.3954	0.0125
E								6188.1536	0.0105
A	6	1	5	←	5	2	4	6225.7760	0.0104
E								6225.0598	-0.0005
A	5	2	4	←	4	2	3	6316.3867	-0.0001
E								6317.0232	-0.0010
A	5	1	5	←	4	0	4	6354.2117	0.0032
E								6361.1953	0.0059
A	9	3	6	←	8	4	4	6372.1322	-0.0015
A	5	4	2	←	4	4	1	6379.0117	-0.0007
E								6379.2553	-0.0009
A	5	4	1	←	4	4	0	6379.4739	-0.0052

E								6379.1643	-0.0031
A	5	3	3	←	4	3	2	6386.5778	0.0017
E								6393.1045	-0.0014
A	9	2	8	←	8	3	6	6391.2280	-0.0142
E								6396.7264	-0.0107
A	9	3	6	←	8	4	5	6399.8112	0.0002
E								6416.8627	0.0004
A	5	3	2	←	4	3	1	6407.0212	0.0054
E								6400.4208	-0.0007
A	11	4	7	←	11	3	9	6422.7148	0.0081
A	11	1	10	←	11	1	11	6448.5764	0.0135
A	11	1	10	←	11	0	11	6456.6150	0.0140
A	3	2	2	←	2	1	2	6492.0843	-0.0019
E								6489.4334	0.0000
A	5	2	3	←	4	2	2	6556.3922	0.0019
E								6555.7744	0.0006
A	3	2	1	←	2	1	2	6561.4757	-0.0044
E								6564.2049	-0.0059
A	4	1	3	←	3	0	3	6565.4227	-0.0062
E								6566.1465	-0.0037
A	5	1	4	←	4	1	3	6588.3966	0.0028
E								6588.1746	0.0024
A	11	2	10	←	11	1	11	6654.3107	-0.0013
A	7	1	6	←	6	2	4	6943.0650	0.0023
A	6	0	6	←	5	1	5	6999.5198	-0.0088
E								6983.2427	0.0033
A	6	1	6	←	5	1	5	7152.8208	0.0028
E								7159.1822	0.0014
A	8	2	6	←	7	3	4	7174.1395	-0.0035
A	4	2	3	←	3	1	2	7195.8367	0.0023
E								7195.1637	-0.0006
A	10	5	6	←	10	4	6	7197.1663	-0.0047
A	10	5	5	←	10	4	6	7206.0276	-0.0094
A	3	2	1	←	2	0	2	7219.3302	-0.0006
E								7222.5598	-0.0032
A	6	0	6	←	5	0	5	7246.5802	0.0068
E								7240.1808	-0.0040
A	12	2	11	←	12	1	12	7299.5094	0.0188
A	9	5	5	←	9	4	5	7307.8591	-0.0057
A	9	5	4	←	9	4	5	7310.8765	0.0014
A	11	5	6	←	11	4	8	7359.9792	0.0026
A	8	2	6	←	7	3	5	7365.9277	-0.0050
E								7367.3166	0.0023
A	8	5	4	←	8	4	4	7375.8082	-0.0009
A	8	5	3	←	8	4	4	7376.6809	0.0001
E								7394.6859	0.0012
A	9	5	5	←	9	4	6	7377.6656	0.0014
A	9	5	4	←	9	4	6	7380.6785	0.0040
A	4	2	2	←	3	1	2	7396.6454	0.0000
E								7397.7074	-0.0037
A	6	1	6	←	5	0	5	7399.8627	-0.0001
E								7416.1340	0.0077
A	8	5	4	←	8	4	5	7403.4822	-0.0042
E								7386.4876	-0.0043
A	7	5	3	←	7	4	3	7416.6557	0.0060
A	7	5	2	←	7	4	3	7416.8563	0.0035
A	7	5	3	←	7	4	4	7426.0606	-0.0083
E								7415.1171	-0.0032
A	7	5	2	←	7	4	4	7426.2657	-0.0063
A	6	5	1	←	6	4	2	7440.8209	-0.0049
E								7449.7534	-0.0004
A	6	5	2	←	6	4	3	7443.3974	0.0014
E								7435.4682	-0.0062
A	5	5	1	←	5	4	1	7454.7169	0.0091
A	5	5	0	←	5	4	1	7454.7169	0.0060
E								7462.6039	0.0002
A	5	5	1	←	5	4	2	7455.2384	0.0053
E								7448.3380	-0.0097
A	5	5	0	←	5	4	2	7455.2384	0.0022
A	6	2	5	←	5	2	4	7554.1655	-0.0060
E								7554.6189	-0.0061
A	6	5	2	←	5	5	1	7652.5544	0.0130
A	6	4	3	←	5	4	2	7664.3782	-0.0003
E								7665.4288	0.0027
A	6	4	2	←	5	4	1	7666.4602	0.0028
E								7665.3258	0.0006
A	6	3	4	←	5	3	3	7669.8481	0.0005
E								7676.1281	-0.0047
A	6	3	3	←	5	3	2	7722.5119	-0.0076
E								7716.1483	-0.0049
A	4	3	1	←	4	0	4	7727.4175	-0.0026
A	7	1	6	←	6	2	5	7752.1880	-0.0064
E								7750.3697	-0.0052
A	6	1	5	←	5	1	4	7852.0736	0.0029
E								7851.7077	0.0021
A	6	2	4	←	5	2	3	7922.4806	-0.0079

E								7922.1659	-0.0072
A	8	1	7	←	7	2	5	7932.5563	0.0110
E								7929.9653	-0.0002
A	4	2	3	←	3	1	3	7947.0066	-0.0006
E								7945.6148	-0.0042
A	3	3	1	←	2	2	0	7961.6241	-0.0023
A	3	3	0	←	2	2	0	7962.6255	-0.0006

E								7974.8597	-0.0015
A	3	3	1	←	2	2	1	7975.7345	-0.0012
E								7964.0129	-0.0029
A	3	3	0	←	2	2	1	7976.7342	-0.0012
								RMS	6.18 kHz

Table S11: Line list for complex 2 (OH–P isomer) for PVE–MeOH.

J‘	K_a‘	K_c‘		J“	K_a“	K_c“	ν_{obs}	ν_{obs}-ν_{calc}
2	1	2	←	1	1	1	2739.1116	-0.0003
2	0	2	←	1	0	1	2874.5870	-0.0001
4	0	4	←	3	2	1	2918.3706	-0.0072
9	0	9	←	8	2	6	3537.2009	-0.0007
5	1	4	←	4	3	1	3727.9913	-0.0002
8	0	8	←	7	2	5	3777.5820	0.0003
3	1	3	←	2	1	2	4084.4973	0.0054
3	0	3	←	2	0	2	4219.0133	-0.0070
3	2	1	←	2	2	0	4532.9681	-0.0024
3	1	2	←	2	1	1	4612.5620	0.0068
6	1	5	←	5	3	2	5025.6624	0.0046
4	1	4	←	3	1	3	5408.9380	0.0124
4	0	4	←	3	0	3	5503.4937	-0.0043
4	1	3	←	3	1	2	6083.6393	-0.0021
4	2	2	←	3	2	1	6131.1840	-0.0004
5	0	5	←	4	0	4	6767.0431	0.0073
5	2	4	←	4	2	3	7198.6402	-0.0099
5	3	3	←	4	3	2	7384.3717	0.0018
5	4	1	←	4	4	0	7394.1856	0.0039
5	2	3	←	4	2	2	7718.2765	-0.0076
							RMS	5.55 kHz

Table S12: Spectroscopic parameters for the OH–O' isomer of PVE–MeOH (complex 1) and its nine heavy atom isotopologues. Distortion for all isotopologues is held fixed to the reported parent species values.

	**N**	**C1**	**C2**	**C3**	**C4**
A(MHz)	1466.59120(26)	1459.794(28)	1460.6056(28)	1465.8070(28)	1460.1496(21)
B(MHz)	697.48965(11)	694.8773(11)	690.87048(74)	688.25516(64)	690.83074(59)
C (MHz)	572.109900(95)	571.0688(11)	567.65107(64)	565.86978(56)	568.31913(49)
Δ_J (kHz)	0.72697(62)	*b*	*b*	*b*	*b*
Δ_{JK} (kHz)	-0.6669(26)	*b*	*b*	*b*	*b*
Δ_K (kHz)	5.6217(62)	*b*	*b*	*b*	*b*
δ_J (kHz)	0.15121(11)	*b*	*b*	*b*	*b*
δ_K (kHz)	2.5783(29)	*b*	*b*	*b*	*b*
N_{lines}[a]	213	19	17	25	20
σ(kHz)	6.68	4.89	8.05	6.39	8.12
	**C5**	**C6**	**C7**	**C8**	**C9**
A(MHz)	1460.5239(17)	1465.7805(97)	1452.9728(46)	1452.9325(23)	1445.44165(90)
B(MHz)	695.72455(46)	697.18509(67)	695.65450(59)	690.36070(41)	692.82176(37)
C (MHz)	570.95295(41)	571.91318(58)	568.84476(42)	565.34500(38)	566.91917(47)
Δ_J (kHz)	*b*	*b*	*b*	*b*	*b*
Δ_{JK} (kHz)	*b*	*b*	*b*	*b*	*b*
Δ_K (kHz)	*b*	*b*	*b*	*b*	*b*
δ_J (kHz)	*b*	*b*	*b*	*b*	*b*
δ_K (kHz)	*b*	*b*	*b*	*b*	*b*
N_{lines}[a]	19	15	17	19	11
σ(kHz)	7.98	8.36	7.00	6.36	8.14

[a] The number of fitted lines.

[b] Kept fixed at the value for the normal species.

[N] Normal species.

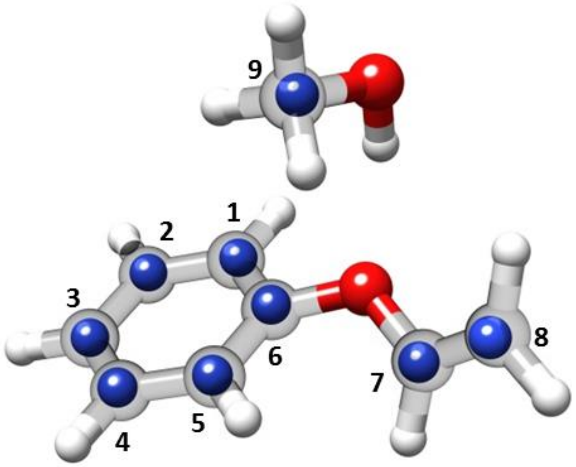

Figure S4: Experimental rotational parameters for the assigned ^{13}C isotopologues for the OH–O’ isomer of PVE–MeOH.

Table S13: Line list of $^{13}C_1$ for the OH–O’ isomer of PVE–MeOH.

J‘	K_a‘	K_c‘		J“	K_a“	K_c“	ν_{obs}	ν_{obs}-ν_{calc}
3	0	3	←	2	0	2	3743.5104	-0.0013
3	1	2	←	2	1	1	3974.2515	-0.0081
4	1	4	←	3	1	3	4791.0624	-0.0044
4	0	4	←	3	0	3	4934.5072	-0.0042
4	1	3	←	3	1	2	5280.3838	-0.0022
5	1	5	←	4	1	4	5968.7326	-0.0064
5	0	5	←	4	0	4	6093.0544	0.0023
5	2	4	←	4	2	3	6298.7191	0.0034
5	3	3	←	4	3	2	6367.6248	-0.0033
5	3	2	←	4	3	1	6387.5604	0.0071
5	2	3	←	4	2	2	6534.5864	0.0069
5	1	4	←	4	1	3	6567.6531	0.0023
6	1	6	←	5	1	5	7137.1517	-0.0013
6	0	6	←	5	0	5	7230.6875	0.0099
6	1	6	←	5	0	5	7384.7250	0.0000
6	2	5	←	5	2	4	7533.4343	-0.0060
6	3	4	←	5	3	3	7647.0675	0.0040
6	1	5	←	5	1	4	7828.2796	-0.0015
6	2	4	←	5	2	3	7895.7956	-0.0034
							RMS	4.89 kHz

Table S14: Line list of $^{13}C_2$ for the OH–O' isomer of PVE–MeOH.

J'	K_a'	K_c'		J"	K_a"	K_c"	ν_{obs}	ν_{obs}-ν_{calc}
3	0	3	←	2	0	2	3722.0137	-0.0056
3	1	2	←	2	1	1	3951.2463	0.0023
4	1	4	←	3	1	3	4762.8915	0.0000
4	0	4	←	3	0	3	4906.5668	0.0076
4	1	3	←	3	1	2	5250.0013	0.0036
4	1	4	←	3	0	3	5283.6285	-0.0142
5	0	5	←	4	0	4	6058.8037	0.0016
5	2	4	←	4	2	3	6262.0405	-0.0053
5	1	4	←	4	1	3	6530.2277	0.0126
6	1	6	←	5	1	5	7095.4460	-0.0042
6	0	6	←	5	0	5	7189.9920	0.0036
6	1	6	←	5	0	5	7347.4942	-0.0009
6	3	4	←	5	3	3	7601.9637	0.0088
7	1	6	←	6	2	5	7660.2915	-0.0179
6	1	5	←	5	1	4	7784.2627	0.0019
6	2	4	←	5	2	3	7848.3751	0.0110
3	3	0	←	2	2	1	7940.9383	-0.0061
							RMS	8.05 kHz

Table S15: Line list of $^{13}C_3$ for the OH–O' isomer of PVE–MeOH.

J'	K_a'	K_c'		J"	K_a"	K_c"	ν_{obs}	ν_{obs}-ν_{calc}
3	1	3	←	2	1	2	3570.8389	0.0054
3	3	1	←	3	2	2	4201.3217	0.0037
4	2	3	←	3	2	2	5005.9465	-0.0005
4	2	2	←	3	2	1	5130.6559	-0.0101
4	1	3	←	3	1	2	5231.4508	-0.0096
4	1	4	←	3	0	3	5276.8530	-0.0033
5	0	5	←	4	1	4	5655.7437	0.0064
5	1	5	←	4	1	4	5914.8587	0.0010
5	0	5	←	4	0	4	6041.0457	-0.0052
5	2	4	←	4	2	3	6240.7262	-0.0032
5	1	5	←	4	0	4	6300.1699	-0.0015
5	3	3	←	4	3	2	6307.3326	-0.0013
5	3	2	←	4	3	1	6326.0585	-0.0037
5	2	3	←	4	2	2	6469.5149	-0.0114
5	1	4	←	4	1	3	6507.8447	-0.0060
6	1	6	←	5	1	5	7073.1570	-0.0011

6	0	6	←	5	0	5	7169.2906	0.0016
6	1	6	←	5	0	5	7332.2869	0.0083
6	2	5	←	5	2	4	7464.7419	0.0007
6	4	3	←	5	4	2	7568.9661	-0.009
6	3	4	←	5	3	3	7574.7510	0.0006
7	1	6	←	6	2	5	7605.2745	0.0065
6	3	3	←	5	3	2	7623.1253	0.0037
6	1	5	←	5	1	4	7758.7550	0.0055
6	2	4	←	5	2	3	7817.7475	0.0161
							RMS	6.39 kHz

Table S16: Line list of $^{13}C_4$ for the OH–O' isomer of PVE–MeOH.

J'	K_a'	K_c'		J"	K_a"	K_c"	ν_{obs}	ν_{obs}-ν_{calc}
3	1	3	←	2	1	2	3585.6225	-0.0066
7	3	5	←	7	2	6	4538.8386	0.0034
4	1	4	←	3	1	3	4767.0672	-0.0005
4	0	4	←	3	0	3	4910.3320	-0.0019
4	2	3	←	3	2	2	5025.9268	0.0037
4	2	2	←	3	2	1	5151.9320	-0.0168
4	1	3	←	3	1	2	5251.4209	-0.0079
5	0	5	←	4	1	4	5685.8947	0.0154
5	1	5	←	4	1	4	5939.1659	-0.0007
5	0	5	←	4	0	4	6064.1035	-0.0024
5	2	4	←	4	2	3	6265.5025	-0.0023
5	3	3	←	4	3	2	6332.8048	-0.0041
5	2	3	←	4	2	2	6496.3512	-0.0061
5	1	4	←	4	1	3	6532.3780	0.0056
6	0	6	←	5	1	5	6943.5597	0.0164
6	1	6	←	5	1	5	7102.1389	-0.0085
6	2	5	←	5	2	4	7494.1987	0.0005
6	3	4	←	5	3	3	7605.2742	-0.0080
6	3	3	←	5	3	2	7654.7099	0.0121
6	1	5	←	5	1	4	7787.4527	0.0020
							RMS	8.12 kHz

Table S17: Line list of $^{13}C_5$ for the OH–O' isomer of PVE–MeOH.

J'	K_a'	K_c'		J"	K_a"	K_c"	ν_{obs}	ν_{obs}-ν_{calc}
3	1	2	←	2	1	1	3977.7526	-0.0022
4	3	2	←	3	3	1	5091.9089	-0.0020
4	3	1	←	3	3	0	5097.8362	-0.0011
4	2	2	←	3	2	1	5186.4411	-0.0138
4	1	3	←	3	1	2	5284.7570	0.0094
5	1	5	←	4	1	4	5969.2748	-0.0003
5	2	4	←	4	2	3	6301.9061	-0.0022
5	3	3	←	4	3	2	6371.7979	-0.0011
5	3	2	←	4	3	1	6392.1562	-0.0104
5	2	3	←	4	2	2	6540.8735	0.0076
5	1	4	←	4	1	3	6572.5748	0.0152
6	1	6	←	5	1	5	7137.4904	-0.0025
6	0	6	←	5	0	5	7230.6875	-0.0068
6	1	6	←	5	0	5	7382.9724	0.0115
6	2	5	←	5	2	4	7536.8977	-0.0055
6	3	4	←	5	3	3	7652.0696	-0.0118
6	1	5	←	5	1	4	7833.2966	0.0116
6	2	4	←	5	2	3	7903.5763	0.0037
3	3	1	←	2	2	0	7929.8501	-0.0023
							RMS	7.98 kHz

Table S18: Line list of $^{13}C_6$ for the OH–O' isomer of PVE–MeOH.

J'	K_a'	K_c'		J"	K_a"	K_c"	ν_{obs}	ν_{obs}-ν_{calc}
3	0	3	←	2	1	2	3094.5435	-0.0082
3	1	3	←	2	1	2	3610.9906	0.0053
4	2	3	←	3	2	2	5065.2496	0.0031
4	2	2	←	3	2	1	5196.5374	0.0081
4	1	3	←	3	1	2	5295.3301	-0.0059
5	1	5	←	4	1	4	5979.9680	0.0104
5	2	4	←	4	2	3	6313.9177	0.0034
5	3	3	←	4	3	2	6384.0274	-0.0045
5	1	4	←	4	1	3	6585.6799	-0.0168
6	1	6	←	5	1	5	7150.2245	0.0019
6	0	6	←	5	0	5	7243.9168	-0.0073
6	2	5	←	5	2	4	7551.2250	-0.0052

6	3	4	←	5	3	3	7666.8039	0.0142
6	1	5	←	5	1	4	7848.8844	-0.0076
6	2	4	←	5	2	3	7919.1997	0.0082
							RMS	8.36 kHz

Table S19: Line list of $^{13}C_7$ for the OH–O’ isomer of PVE–MeOH.

J‘	K_a‘	K_c‘		J“	K_a“	K_c“	ν_{obs}	ν_{obs}-ν_{calc}
4	0	4	←	3	1	3	4418.4338	0.0141
4	1	4	←	3	1	3	4777.9483	0.0034
4	2	2	←	3	2	1	5182.2083	0.0019
4	1	3	←	3	1	2	5278.7551	0.0072
5	1	5	←	4	1	4	5951.3811	0.0008
5	0	5	←	4	0	4	6074.5524	0.0071
5	2	4	←	4	2	3	6289.7435	-0.0021
5	1	5	←	4	0	4	6310.8984	-0.0070
5	3	3	←	4	3	2	6362.2597	-0.0122
5	3	2	←	4	3	1	6383.9621	-0.0004
5	2	3	←	4	2	2	6536.8359	0.0109
5	1	4	←	4	1	3	6563.5316	-0.0004
6	1	6	←	5	1	5	7115.2362	-0.0085
6	0	6	←	5	0	5	7206.4939	0.0070
6	2	5	←	5	2	4	7521.3022	-0.0011
6	3	4	←	5	3	3	7640.6210	-0.0047
7	1	6	←	6	2	5	7753.1641	-0.0065
							RMS	7.00 kHz

Table S20: Line list of $^{13}C_8$ for the OH–O’ isomer of PVE–MeOH.

J‘	K_a‘	K_c‘		J“	K_a“	K_c“	ν_{obs}	ν_{obs}-ν_{calc}
3	0	3	←	2	0	2	3711.6344	-0.0070
4	1	4	←	3	1	3	4747.1734	-0.0057
4	0	4	←	3	0	3	4890.9545	0.0000
4	2	3	←	3	2	2	5011.6475	0.0078
4	1	3	←	3	1	2	5241.1352	0.0032
5	1	5	←	4	1	4	5913.5086	-0.0012
5	0	5	←	4	0	4	6037.4913	0.0012
5	1	5	←	4	0	4	6280.9859	0.0101

5	3	3	←	4	3	2	6317.1416	-0.0086
5	3	2	←	4	3	1	6337.7319	-0.0053
5	2	3	←	4	2	2	6487.0561	0.0047
5	1	4	←	4	1	3	6517.8019	0.0064
4	2	3	←	3	1	2	7121.2135	-0.0021
6	0	6	←	5	0	5	7163.1937	-0.0023
6	2	5	←	5	2	4	7470.6732	-0.0010
6	4	2	←	5	4	1	7583.2063	-0.0147
6	3	4	←	5	3	3	7586.5035	0.0103
6	3	3	←	5	3	2	7639.5263	0.0018
7	1	6	←	6	2	5	7668.6538	0.0009
							RMS	6.36 kHz

Table S21: Line list of $^{13}C_9$ for the OH–O’ isomer of PVE–MeOH.

J‘	K_a‘	K_c‘		J“	K_a“	K_c“	ν_{obs}	ν_{obs}-ν_{calc}
3	1	3	←	2	1	2	3581.7346	0.0023
4	3	2	←	4	2	3	4123.5672	0.0084
4	0	4	←	3	0	3	4904.0298	0.0128
5	0	5	←	4	1	4	5695.1525	0.0098
3	3	0	←	3	1	3	6920.0748	-0.0046
6	1	6	←	5	1	5	7090.1220	-0.0045
6	0	6	←	5	0	5	7180.8262	-0.0157
8	2	6	←	7	3	5	7384.2544	-0.0046
6	1	5	←	5	1	4	7789.8878	0.0050
3	3	0	←	2	2	1	7866.3332	-0.0043
6	2	4	←	5	2	3	7868.0155	0.0056
							RMS	8.14 kHz

Table S22: Structures used in the LCCSD(T0)-F12 single-point calculations. All structures have been optimized at the B3LYP-D3(BJ)/def2-TZVP level of theory as described in the main text. The respective x,y,z coordinates are given in Å.

OH–P dimer

C	-0.04836860659466	-0.12588517223625	0.21164999136292
C	-0.02754690957082	-0.22724043749683	1.59487574238489
C	1.17837746319235	-0.18493475103149	2.28948368767373
C	2.36580480592832	-0.01939439508258	1.58675368808279
C	2.35908238541217	0.09269311114876	0.19903629372306
C	1.14724741750355	0.02398175679512	-0.48560409286754
O	1.06229458442829	0.12180509383792	-1.84860106170259

C	2.11817817952707	-0.33238489715085	-2.60341983997592
C	2.40218430714440	0.20750185524794	-3.77776033432761
H	-0.97527402431275	-0.16993494891006	-0.34451614071059
H	-0.95867078250771	-0.35033428614232	2.13321730405456
H	1.19087444261414	-0.27182404432368	3.36795257869123
H	3.30841047600278	0.03052834470414	2.11703199405515
H	3.28113159496744	0.24042087709542	-0.34631856432114
H	2.63629982655632	-1.18871887017599	-2.18453074266500
H	1.85516846641950	1.06032098905557	-4.15637063604047
H	3.18563024585294	-0.21924753806949	-4.38646456406127
O	2.43661379775349	-3.06017572420512	-0.46277399735173
C	1.13441261202449	-3.59199184668711	-0.66864534482982
H	2.36708948769181	-2.30714315737813	0.13664347177458
H	1.24051115337240	-4.44160273925495	-1.34229759140167
H	0.46180347545918	-2.86054438809165	-1.13030456210476
H	0.68332460113532	-3.94041583164836	0.26626172055720

OH–P’ dimer

C	0.46362183152705	-0.86233007964380	-1.31727593526035
C	1.75681706345502	-1.25902699087321	-1.01290809369287
C	2.15966822149107	-1.39760040800295	0.31400946413599
C	1.25631865444989	-1.12396799963905	1.33385342761300
C	-0.04190943138139	-0.71576940794726	1.04223576260352
C	-0.43496570259569	-0.59670568879249	-0.28748361812602
O	-1.68004365371432	-0.16057106300360	-0.66031058185153
C	-2.73532718363995	-0.36566068651642	0.18785287693398
C	-3.78457444964597	0.44113821752064	0.19677521745364
H	0.13432035621685	-0.74666917743586	-2.34117994439219
H	2.45554410110914	-1.45854609889154	-1.81484951904614
H	3.16969499719931	-1.70492939071570	0.54861566097057
H	1.56518760493509	-1.20725504376483	2.36789496919719
H	-0.72727879011275	-0.46365439141135	1.83908388835240
H	-2.66183182687864	-1.25852727233451	0.79990361718058
H	-3.82217089113631	1.32595805505095	-0.42381316935135
H	-4.63414666082205	0.20845040763130	0.82137200758075
O	2.14040415629732	2.07949594524163	0.22468079992319
C	0.87886950494716	2.63449368623346	-0.11132302198874
H	2.05820265311104	1.11912346239724	0.25423702801289
H	1.00400815047455	3.71718670989968	-0.13832822686123
H	0.10969318463689	2.39079906618284	0.63079809258730
H	0.52831891007668	2.29902074881482	-1.09355930197456

OH–O dimer

C	-0.87266873781654	-1.08784260834588	0.31206014672733
C	-2.18092070846638	-1.51772373008050	0.13980136634569
C	-0.58443802450880	0.26745087223693	0.19221192035633

O	0.72771169252552	0.64658531342275	0.40416394782329
C	-1.58487547023678	1.19150029429107	-0.08580733268318
C	-2.88975882322256	0.74259188326135	-0.26416480210399
C	-3.19264303699578	-0.60835105268495	-0.15449508633767
H	-4.20976051516156	-0.95096403310327	-0.29120255197582
H	-2.40867172916600	-2.57208369317436	0.23152473814919
H	-0.07348326975196	-1.78244591547480	0.53655224322112
H	-3.67182397787449	1.45939214410576	-0.47973614943555
C	1.21981726078511	1.73707146674667	-0.26671511496434
C	2.25739411962587	2.41866854505430	0.19078251659109
H	0.72671657240693	1.94968991937253	-1.20845218669611
H	2.72003121449279	2.17942533900213	1.13849950468651
H	2.66696447007888	3.22676319574074	-0.39663045135881
H	-1.35500398728223	2.24705375190958	-0.14046339346285
C	2.74909456634771	-1.72142632818879	-0.81634845287166
H	1.81895478825743	-1.65555055400877	-1.39331924479023
O	2.51299014714199	-1.66774833216877	0.58091936386286
H	3.42306188907144	-0.92396025174247	-1.15031546384959
H	3.21845473634453	-2.68202995238261	-1.02933187275340
H	2.05564032340488	-0.83747227378866	0.76739245551981

OH–O’ dimer

C	1.25847109013911	0.42836912961783	1.22065882941952
C	2.55985431865344	0.75569905602739	0.87022785722598
C	3.10884359520458	0.28918909529061	-0.32115672347579
C	2.34035294147384	-0.50032689722049	-1.16618443504861
C	1.02992587103309	-0.82900006297323	-0.83303652108419
C	0.50269559879281	-0.36743527683190	0.36713053564377
O	-0.79875727583598	-0.61666409287440	0.75274333636218
C	-1.43135575790648	-1.73756633331753	0.26897810247391
C	-2.73131612794076	-1.74585365396792	0.02561066073448
H	0.81619501841300	0.77959859853723	2.14312616512012
H	3.14932206814189	1.37529041103641	1.53402470686498
H	4.12500740013322	0.54528238802268	-0.58929448768615
H	2.75290013365011	-0.85637584911285	-2.10135813417035
H	0.42114523463434	-1.41249165983026	-1.50982135295952
H	-0.78761867811800	-2.60342729032718	0.15964223968061
H	-3.20968817903995	-2.66525571394570	-0.27823654763230
H	-3.33510577286148	-0.85463242545982	0.13598986043434
O	-2.39620180036476	1.78047399397076	0.25090483838483
C	-1.87047218075411	1.94330610416541	-1.05710164285893
H	-1.91260638041651	1.05609613149722	0.66804571743907
H	-2.42425378231121	2.75422183372406	-1.52988183229332
H	-0.80737931561249	2.20860702026491	-1.04325836138398
H	-1.99455531910771	1.03870909370679	-1.66486171119063

OH–E dimer

C	-1.88529345292590	-1.08474857718528	0.67647466405697
C	-3.06071846545818	-0.37160281979420	0.48635105861109
C	-0.70198010064005	-0.60275686340767	0.13030133623568
O	0.44243202221545	-1.33673464717677	0.38305726382804
C	-0.67353914712706	0.58629217933388	-0.58952303674007
C	-1.86201382644617	1.28480240833775	-0.77806903018684
C	-3.05515499740331	0.81140376511417	-0.24681859531858
H	-3.97405219671330	1.36315498500590	-0.39543087354434
H	-3.98514922156903	-0.74502961865383	0.90770958879101
H	-1.86816324921721	-2.01010563948532	1.23639257371748
H	-1.84552560223390	2.21366931747727	-1.33368828761263
C	1.38248004808978	-1.43776180525011	-0.59412784605671
C	2.62989836242229	-1.81155686026668	-0.33027758727027
H	1.02958709828441	-1.21912193074934	-1.59661895951244
H	2.95409204295387	-2.02371292966041	0.67958189431881
H	3.32808786185105	-1.95346410022801	-1.14187162879046
H	0.26386189670181	0.98136957716876	-0.95906867329246
C	2.31323984442125	1.84864019844693	1.02128855132052
H	1.47374095999698	1.27159468583366	1.42370171696246
O	2.56400160448324	1.54102096129251	-0.34373203829714
H	3.19478543511554	1.67326538368741	1.64817351812828
H	2.06102775424058	2.90756472707113	1.07035882061609
H	2.75020622895790	0.59410010308827	-0.40574622996447

OH–E’ dimer

C	2.30029567950477	-1.04496202348354	-0.02914303936444
C	3.32012838504592	-0.10800544290000	0.05617872365762
C	0.98098167962161	-0.61136625672528	-0.08338433730431
O	0.01111134610645	-1.58941660691413	-0.20423562947695
C	0.66765182241499	0.74315866507983	-0.06713090707059
C	1.70188356052773	1.66922967698374	0.02709856949223
C	3.02548818707236	1.25200464858192	0.09133226232698
H	3.82356417758835	1.97955699018359	0.16006748921514
H	4.34872934437613	-0.44268550900789	0.10077010608176
H	2.50744469593791	-2.10657349633299	-0.05272413698794
H	1.46387621605584	2.72549210866663	0.03562328337920
C	-1.17964901296912	-1.40216168483492	0.42232453498755
C	-2.28612606498641	-2.02695779275911	0.03436293552239
H	-1.14723351762993	-0.73458272245699	1.27770697572217
H	-2.29367240128167	-2.68343648898812	-0.82511067163988
H	-3.19120246584675	-1.91818590759863	0.61414115707711
H	-0.35868475572389	1.07449349393307	-0.16544725913930
C	-3.50727522542595	1.52298468412345	0.40282930826137
H	-4.52455168006672	1.12879075084852	0.30250856817177
O	-2.70428031614699	1.21991726803968	-0.72700091556889

H	-3.07337674999022	1.13731301142773	1.33352667897277
H	-3.56422222914608	2.60862491607661	0.47552000024115
H	-2.61764037503833	0.25839211805682	-0.78582909655693
